# Isocyanate‐Modified Melatplatin(IV) Enhances Antitumor Efficacy via TCF4/COL6A3‐Mediated Extracellular Matrix Remodeling and cGAS‐STING Pathway Activation in Bladder Cancer

**DOI:** 10.1002/advs.77833

**Published:** 2026-09-27

**Authors:** Linhui Wang, Xiaomeng Liu, Tao Zhu, Zirun Su, Ruiping Liu, Zhe Li, Kaixuan Du, Kaipeng Jia, Chong Fu, Yuda Lin, Zihan Xue, Zhe Zhang, Chong Shen, Yunkai Qie, Zhouliang Wu, Zhiheng Liu, Kangkang Liu, Jingyuan Xu, Hailong Hu

**Affiliations:** ^1^ Department of Urology The Second Hospital of Tianjin Medical University Tianjin China; ^2^ Tianjin Key Laboratory of Urology Tianjin Institute of Urology The Second Hospital of Tianjin Medical University Tianjin China; ^3^ Tianjin Key Laboratory on Technologies Enabling Development of Clinical Therapeutics and Diagnostics (Theranostics) School of Pharmacy Tianjin Medical University Tianjin China

**Keywords:** bladder cancer, cGAS‐STING pathway, collagen VI, immune regulation, melatonin, platinum(IV) prodrug

## Abstract

Cisplatin (CDDP) chemosensitivity and severe toxicity severely limit its clinical application in muscle‐invasive bladder cancer (MIBC) and non‐muscle‐invasive bladder cancer (NMIBC). To address these unmet clinical needs, we synthesized a series of melatonin‐conjugated platinum(IV) prodrugs functionalized with isocyanate chains (compounds **5**‒**12**), and identified MP‐12C (compound **11**) as the lead candidate with optimal potency and safety. In vitro assays showed that MP‐12C exhibited far stronger antiproliferative activity than CDDP and mitomycin C in parental and CDDP‐resistant bladder cancer cells. Multiple in vivo models, including subcutaneous xenograft, orthotopic, and chemically induced spontaneous bladder cancer models, were adopted. Both intravenous injection and intravesical instillation of MP‐12C achieved robust tumor suppression while reducing toxicity compared to conventional chemotherapeutics. Mechanistically, MP‐12C downregulated the *TCF4/COL6A3* axis to suppress collagen VI secretion by bladder cancer cells, thereby remodeling the extracellular matrix (ECM) and enhancing the sensitivity of bladder cancer cells to CDDP. MP‐12C triggered immunogenic cell death and persistently activated the cGAS‐STING pathway, promoting dendritic cell maturation and CD8^+^ T cell infiltration to remodel the antitumor immune microenvironment. Collectively, MP‐12C integrates ECM modulation and immune activation to exert superior anti‐bladder cancer efficacy with favorable safety, thus providing a promising platinum(IV) prodrug strategy for NMIBC and MIBC.

## Graphical abstract

A series of melatonin‐conjugated platinum(IV) prodrugs was functionalized with isocyanate chains (compounds **5**‒**12**). MP‐12C (compound **11**) was identified as the lead candidate, with optimal potency and safety. MP‐12C exhibited greater efficacy than cisplatin by increasing intracellular uptake, reshaping the extracellular matrix, activating the cGAS‐STING pathway, and inducing ICD to remodel the tumor immune microenvironment.

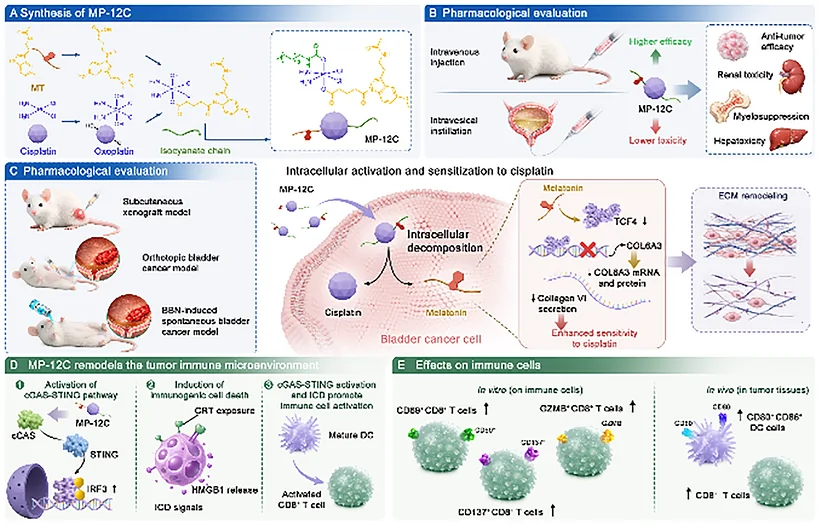

## 1 Introduction

Bladder cancer has the highest incidence rate among all malignant tumors of the urinary system and is the ninth most common cancer worldwide [1]. Based on tumor infiltration, it is primarily categorized into non‐muscle‐invasive bladder cancer (NMIBC) and muscle‐invasive bladder cancer (MIBC); the primary conventional treatment for MIBC and metastatic bladder cancer is cisplatin (CDDP)‐based chemotherapy, while the principal adjuvant therapy for postoperative patient with NMIBC is intravesical instillation chemotherapy or Bacillus Calmette‐Guérin (BCG) [2, 3, 4]. Nonetheless, in actual practice, three major unresolved clinical bottlenecks remain in the treatment of bladder cancer: first, CDDP exhibits significant dose‐limiting toxicities, including irreversible renal injury and bone marrow suppression [5, 6]; Second, approximately 30% of patients are forced to discontinue treatment due to an inability to endure full‐dose chemotherapy [7]. Finally, intrinsic and acquired CDDP resistance are highly prevalent, with nearly 50% of patients with MIBC experiencing disease progression after chemotherapy, and the 5‐year overall survival rate for advanced bladder cancer is below 15% [8]. Additionally, despite patients with NMIBC undergoing transurethral bladder tumor resection followed by intravesical instillation chemotherapy or BCG, the recurrence and progression rates remain elevated [9, 10]. Consequently, the development of novel integrated pharmaceuticals that exhibit significant antitumor efficacy, minimal systemic toxicity, the ability to overcome chemotherapy resistance, reduced recurrence and progression rates, and the capacity to modify the tumor immune microenvironment is a critical scientific challenge that must be urgently addressed in the clinical management of bladder cancer.

Melatonin (MT) is an endogenous hormone produced by the pineal gland that exhibits remarkable biological safety and lacks dose‐limiting toxicity. It has been extensively used in the therapeutic context to enhance sleep and mitigate oxidative stress [11, 12, 13]. Prior research has established that MT possesses antitumor properties by modulating the cell cycle, apoptotic pathways, and oxidative stress, among other mechanisms. It also demonstrates notable chemotherapy sensitization, anti‐inflammatory, and immune regulatory effects, rendering it an optimal functional ligand for the structural modification of platinum‐based drugs [14, 15, 16]. Nevertheless, current research primarily involves the co‐administration of MT and CDDP, which presents challenges owing to discrepancies in the pharmacokinetic profiles of the two agents and the difficulty in achieving simultaneous delivery to tumor tissues, complicating the optimization of their synergistic effects and highlighting a distinct opportunity for the development of novel platinum(IV) prodrugs [17, 18].

Platinum(IV) prodrugs are the primary focus of contemporary research on platinum‐based antitumor agents and are a significant tool for addressing the clinical shortcomings of conventional CDDP. In contrast to the planar quadrilateral configuration of the traditional CDDP (platinum(II)), platinum(IV) prodrugs exhibit stable octahedral spatial structures that are resistant to degradation under neutral physiological conditions. This stability significantly reduces nonspecific drug exposure and systemic toxicity in normal tissues. Upon entry of platinum(IV) prodrugs into tumor cells, they are reduced to the active platinum(II) core within the tumor microenvironment by high concentrations of reducing agents, simultaneously releasing axially modified functional ligands. This process facilitates tumor‐targeted delivery, multi‐targeted synergistic enhancement, reduced toxicity, and sensitization [19, 20]. Previous studies have established that altering the axial functional ligand of platinum(IV) can effectively counteract tumor resistance and enhance antitumor efficacy [21]. However, current platinum(IV) prodrugs for bladder cancer mainly focus on direct cytotoxicity, whereas a few candidates simultaneously target the extracellular matrix (ECM) barrier associated with chemotherapeutic drug sensitivity and the immunosuppressive tumor microenvironment. Notably, *COL6A3*‐mediated collagen VI deposition is a key driver of bladder cancer chemosensitivity; however, no platinum‐based agents have been reported to regulate this axis. In addition, conventional cisplatin fails to sustain cGAS‐STING activation and may damage immune cells, thereby limiting its immunotherapeutic potential.

**SCHEME 1:**
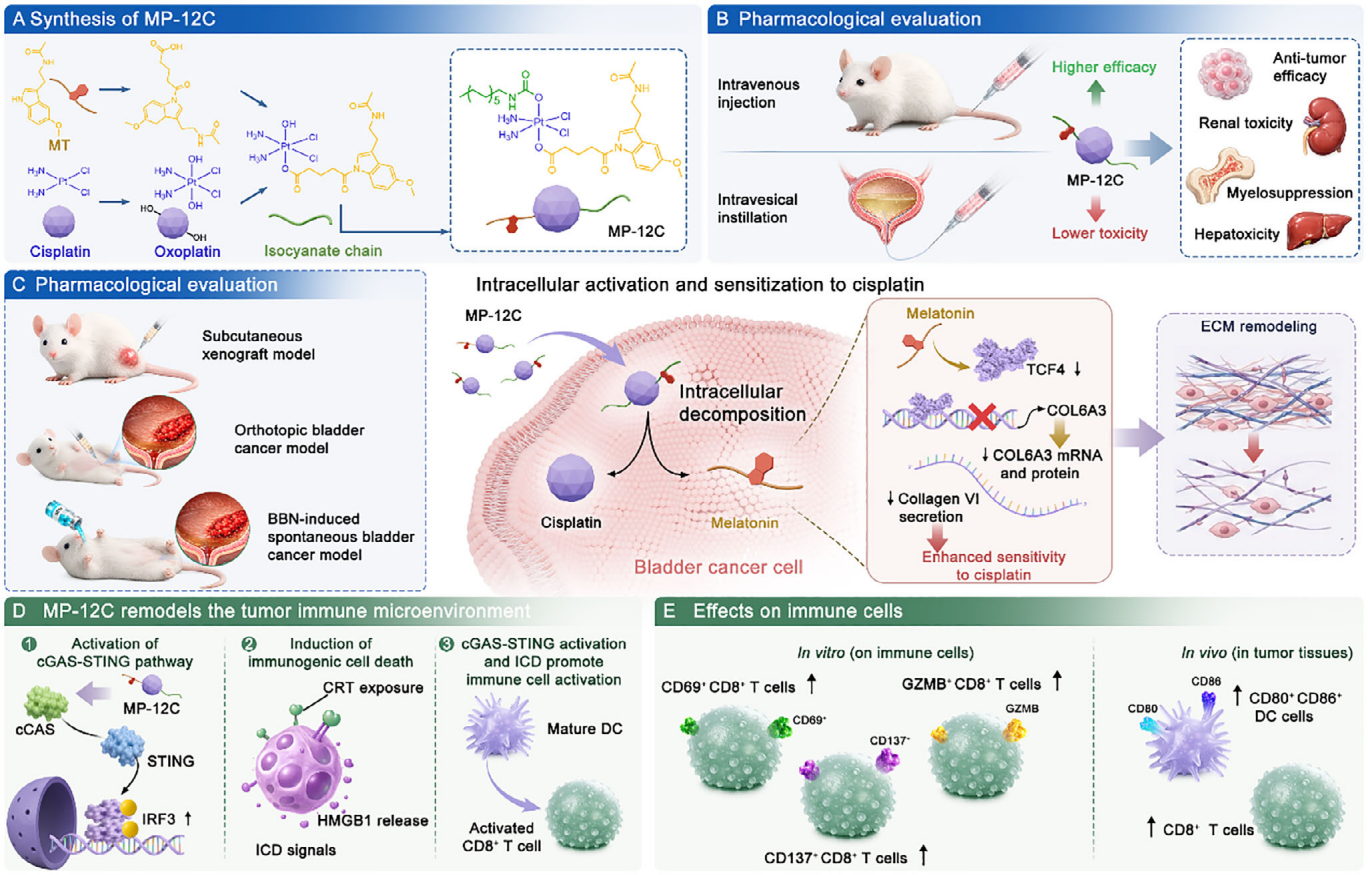
The schematic illustration of MP‐12C with higher efficiency and lower toxicity. A novel melatonin/isocyanate dual‐modified platinum(IV) prodrug, MP‐12C exerts potent, low‐toxicity anti‐bladder cancer effects compared with cisplatin and mitomycin C. MP‐12C simultaneously remodels the extracellular matrix via TCF4/COL6A3/collagen VI axis inhibition, and activates the cGAS‐STING pathway, induces ICD, drives dendritic cell maturation and CD8^+^ T cell infiltration to reshape the tumor immune microenvironment.

In this study, we designed and synthesized a series of melatonin/isocyanate double‐modified platinum (IV) prodrugs (compounds **5‒12**), and hypothesized that the best candidate MP‐12C, can overcome the resistance of bladder cancer cells to CDDP, remodel the ECM through TCF4/COL6A3/collagen VI to enhance the drug sensitivity of bladder cancer cells to CDDP, induce immunogenic cell death (ICD), and activate cGAS‐STING to remodel the tumor immune microenvironment. We then systematically evaluated the efficacy, biosafety, and mechanism of action across two routes of administration (intravenous injection and intravesical instillation) in both in vitro and in vivo models (subcutaneous xenograft, orthotopic, and chemically induced spontaneous bladder cancer). This study aimed to identify a new preclinical candidate drug and a multi‐target treatment strategy for NMIBC and MIBC (Scheme 1).

## 2 Results

### 2.1 Synthesis and Characterization of Melatplatin(IV) Prodrugs 5‒12

**FIGURE 1:**
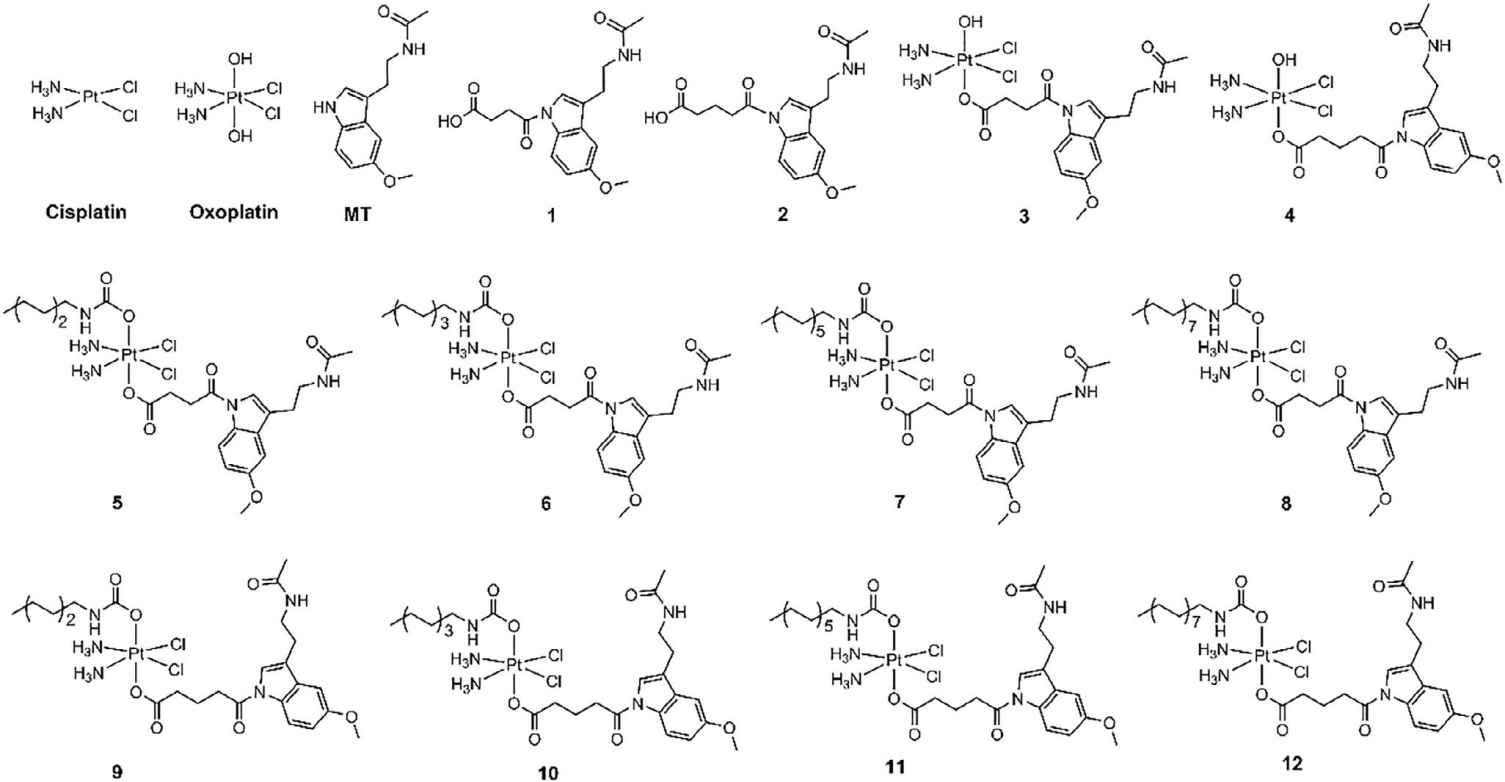
The structures of CDDP, oxoplatin, MT, and its derivatives **1** and **2**, and platinum(IV) prodrugs **3**‒**12**.

**SCHEME 2:**
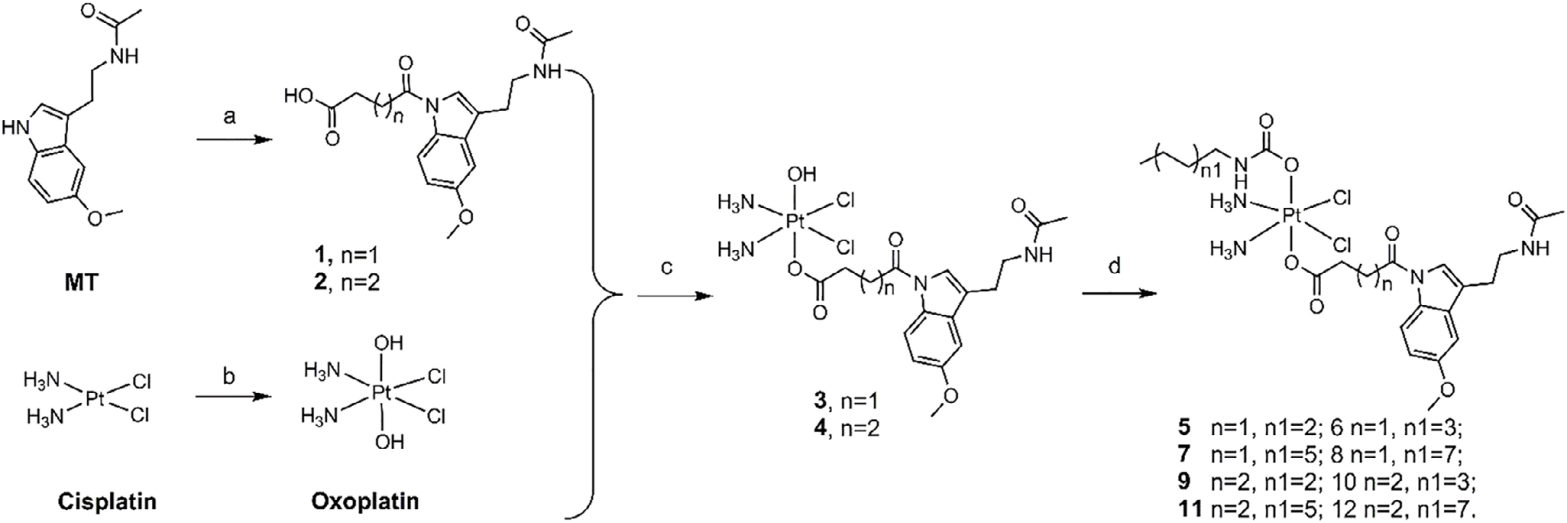
Synthesis flowchart of various platinum(IV) prodrugs. (a) Succinic anhydride or glutaric anhydride, acetonitrile, TEA, DMAP, 50°C, overnight. (b) H2O2 (30%), 5 h, 70°C. (c) TBTU, TEA, DMSO, room temperature, overnight. (d) Hexyl isocyanate, octyl isocyanate, dodecyl isocyanate, or hexadecyl isocyanate, 30°C, 5 h.

Eight novel melatplatin(IV) compounds **5**‒**12** were first designed and synthesized (Figure 1) by introducing MT and isocyanate in the axial positions of the platinum(IV) center; the synthetic routes are shown in Scheme 2. Compounds **1**‒**4** and *c,c,t‐[Pt(NH3)_2_Cl_2_(OH)_2_]* (oxoplatin) were synthesized as described previously [22, 23]. Compounds **1** and **2** were synthesized by amidating MT with either succinic anhydride or glutaric anhydride, and oxoplatin was synthesized by oxidizing CDDP with hydrogen peroxide (H_2_O_2_ 30%). Compounds **3** and **4** were obtained by simple esterification of compounds **1** and **2** with oxoplatin in the presence of 2‐(1H‐benzotriazole‐1‐yl)‐1,1,3,3‐tetramethyluroniumtetrafluoro borate (TBTU), triethylamine (TEA), and dimethyl sulfoxide (DMSO). Compounds **5**‒**12** were synthesized by esterifying compounds **3** or **4** with isocyanate in N‐dimethylformamide (DMF). The structures of all the compounds were characterized by ^1^H/^13^C Nuclear Magnetic Resonance (NMR) and High Resolution Mass Spectrometry (HR‐MS). The purity over 95% and the stability of the compounds in phosphate‐buffered saline (PBS) were monitored by HPLC. All data are shown in Figures S1–S49.

### 2.2 Cytotoxicity Profiles of Various Compounds

The cytotoxicity of the compounds was evaluated using the MTT assay in four cancer cell lines: HepG‐2 (hepatocellular carcinoma), MCF‐7 (breast carcinoma), HeLa (cervical carcinoma), and A549 (non‐small cell lung carcinoma), as well as a human normal liver cell line (HUVEC). The half‐maximal inhibitory concentration (IC_50_) values after 72 h of treatment are reported in Table 1. CDDP, oxaliplatin, MT, and a combination of CDDP and MT (mixture; MT+CDDP) were used as positive controls. These results indicated that the cytotoxicity of compounds **5–12** was greater than that of CDDP. Among them, compound **11** (MP‐12C) exhibited optimal antiproliferative effects and the lowest toxicity compared with CDDP and the combination group across all tested cell lines. MP‐12C was 87‐fold more potent than CDDP in MCF‐7 cells (IC_50_ = 0.068 ± 0.004 µm vs. 5.93 ± 0.40 µm for CDDP). In addition, the cytotoxicity of MP‐12C in HUVECs was evaluated and compared with that of normal HUVECs. The selectivity index of MP‐12C for tumor cells was 3.19, whereas that of cisplatin was only 0.46, indicating MP‐12C's potential to reduce toxicity.

**TABLE 1:** Cell cytotoxicity (IC_50_ in µm) of MT and the platinum compounds for 72 h.

Compounds	HepG‐2	MCF‐7	HeLa	A549	HUVEC	SI^c^
CDDP	1.62 ± 0.02	5.93 ± 0.40	4.94 ± 0.31	3.11 ± 1.17	2.73 ± 0.77	0.46
Mixture	1.43 ± 0.42	3.03 ± 0.12	3.69 ± 0.96	4.21±0.75	2.77 ± 0.34	1.94
MT	73.92 ± 23.03	16.49 ± 0.91	60.27 ± 1.71	60.04 ± 24.19	> 100	‒
**5**	0.283 ± 0.149	0.511 ± 0.064	0.688 ± 0.111	0.799 ± 0.133	0.139 ± 0.081	0.27
**6**	0.252 ±0.007	0.148 ± 0.014	0.561 ± 0.056	0.224 ± 0.037	0.128 ±0.024	0.86
**7**	0.143 ± 0.011	0.195 ± 0.083	0.147 ± 0.018	0.247 ± 0.013	0.342 ± 0.007	1.75
**8**	0.295 ± 0.046	0.595 ± 0.044	0.088 ± 0.033	0.231 ± 0.094	0.065 ± 0.021	0.11
**9**	0.167 ± 0.066	0.523 ± 0.093	0.549 ± 0.206	0.337 ± 0.039	0.214 ± 0.008	0.41
**10**	0.175 ± 0.018	0.090 ± 0.004	0.434 ± 0.006	0.107 ± 0.007	0.189 ± 0.008	2.1
**11**	0.123 ± 0.008	0.068 ± 0.004	0.161 ± 0.012	0.343 ± 0.034	0.217 ± 0.025	3.19
**12**	0.061 ± 0.004	0.536 ± 0.089	0.282 ± 0.137	0.281 ± 0.024	0.176 ± 0.071	0.33
FI^a^	13.17	87.21	30.68	9.07	12.58	
FI^b^	11.63	44.56	22.92	12.27	12.76	

^a^ FI, fold increase; IC_50_ (CDDP)/ IC_50_ (compound **11**);

^b^ IC_50_ (mixture)/ IC_50_ (compound **11**);

^c^ SI, selectivity index; IC_50_ (in HUVEC)/ IC_50_ (in MCF‐7 cells).

### 2.3 MT Sensitizes Bladder Cancer Cells to CDDP

The plate colony formation assay results indicated that the colony size of T24 bladder cancer cells in the MT group was slightly reduced compared to that in the control group. The colony size of T24 cells in the CDDP group was significantly reduced, indicating that CDDP alone significantly impaired the colony‐forming capacity of T24 bladder cancer cells, thereby inhibiting tumor cell proliferation. The colony size produced by T24 cells in the MT+CDDP group was dramatically reduced compared to that in the CDDP group, and it decreased further. This suggests that MT augments CDDP's inhibitory effect on T24 bladder cancer cell proliferation, indicating that MT sensitizes these cells to CDDP. The concomitant application of both agents substantially reduced T24 bladder cancer cells’ colony‐forming capacity, demonstrating a pronounced synergistic inhibitory effect (Figure S50A,B). Subsequently, a 5‐ethynyl‐2'‐deoxyuridine (EdU) proliferation assay was conducted to verify whether MT enhanced CDDP's antiproliferative effect on bladder cancer cells. The treatment conditions for each cell group were aligned with those used in the plate colony formation experiment and were categorized as control, MT, CDDP, and MT+CDDP. The results indicated that treatment with a low dose of MT alone did not significantly affect the proliferation of T24 bladder cancer cells. In contrast, treatment with CDDP alone significantly inhibited cell proliferation. Additionally, the MT+CDDP combination markedly enhanced CDDP's inhibitory effect on T24 bladder cancer cell proliferation, indicating that MT sensitized the cells to CDDP. The concurrent application of both agents significantly reduced the proliferative capacity of T24 bladder cancer cells, reinforcing the conclusions drawn from the plate colony formation experiments (Figure S50C,D). These results supported the rationale for constructing melatonin‐conjugated platinum prodrugs.

### 2.4 In Vitro Antitumor Efficacy of MP‐12C

**FIGURE 2:**
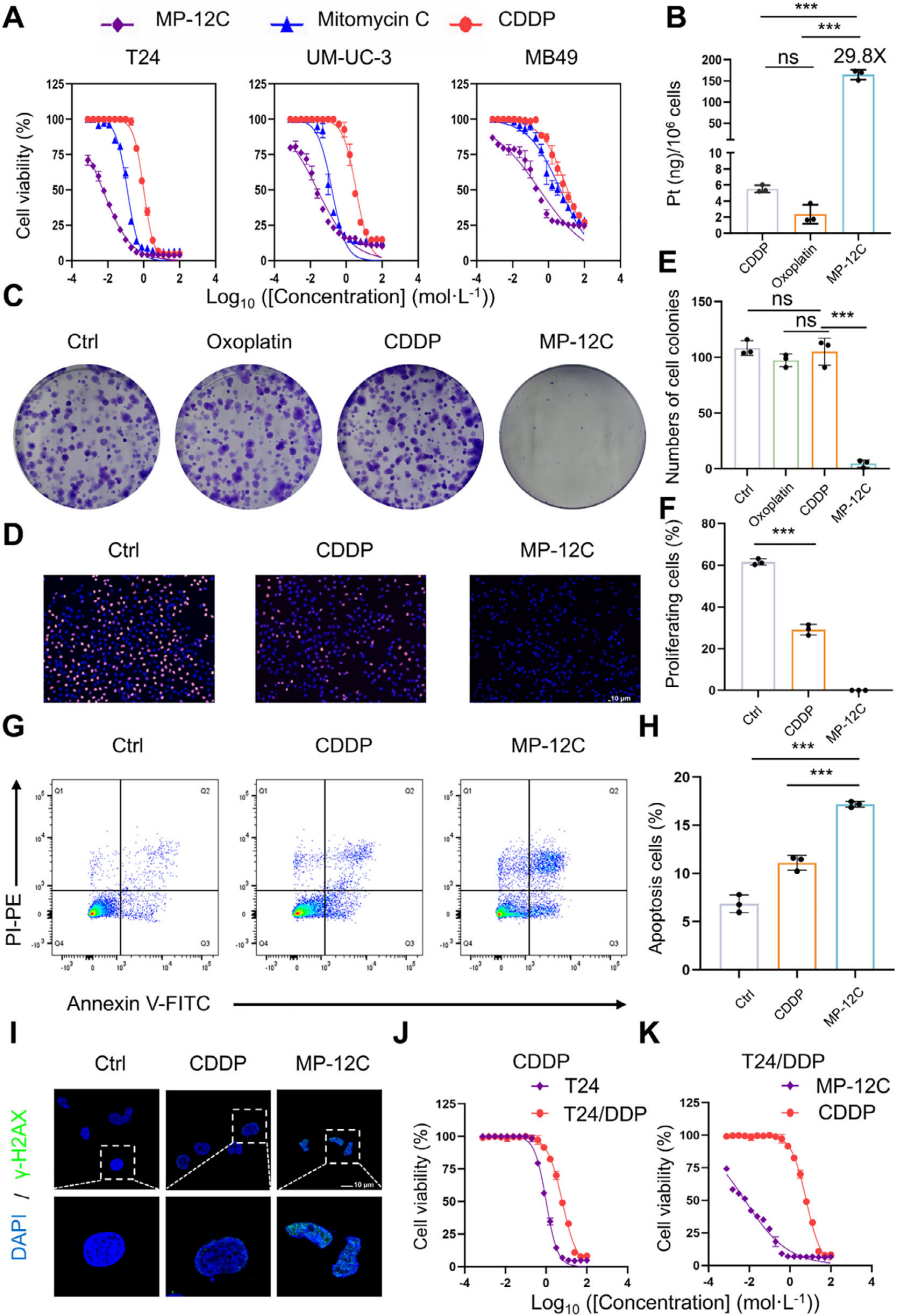
The results of in vitro experiments on the effects of different drugs on bladder cancer. (A) The IC_50_ values of CDDP, MP‐12C, and mitomycin C in T24 bladder cancer cells were determined using the CCK‐8 Cell Viability Assay (n = 3). (B) The platinum accumulation in T24 bladder cancer cells was identified using ICP‐MS. (C) The inhibitory effect of oxoplatin, CDDP, and MP‐12C on the proliferation of T24 bladder cancer cells was detected by a plate cloning assay. (D) EdU Cell Proliferation Assay to assess the inhibitory impact of CDDP and MP‐12C on the proliferation of T24 bladder cancer cells. (E) Statistical analysis of cell colony number generated by the plate cloning assay (n = 3). (F) Statistical examination of the proportion of EdU proliferating cells to the total cell count (n = 3). (G) Detection of apoptosis in T24 bladder cancer cells following treatment with CDDP and MP‐12C using flow cytometry. (H) Statistical examination of the rate of cell apoptosis (n = 3). (I) Immunofluorescence labeling was employed to assess the extent of DNA damage in T24 bladder cancer cells subjected to CDDP and MP‐12C, utilizing γ‐H2AX as a marker for DNA double‐strand breaks. (J) The CCK‐8 Cell Viability Assay was used to measure the IC_50_ of CDDP in both parental T24 and T24/DDP cells. (K) The CCK‐8 Cell Viability Assay was used to measure the IC_50_ of CDDP and MP‐12C in T24/DDP cells. Data are presented as mean ± SD. Statistical analysis was performed using one‐way ANOVA followed by Tukey's post‐hoc test (B, E, H). Statistical analysis was performed using a two‐sided Student's *t*‐test (F). ^*^ *p* < 0.05, ^**^ *p* < 0.01, ^***^ *p* < 0.001; ns, not significant.

The drug's in vitro inhibitory effect on bladder cancer cell growth was assessed using the CCK‐8 assay. The findings indicated that the human bladder cancer cell lines T24 and UM‐UC‐3 and the murine bladder cancer cell lines MB49 and MP‐12C demonstrated markedly enhanced antitumor proliferation efficacy relative to CDDP and the frequently used intravesical instillation agent, mitomycin C. In two human bladder cancer cell lines (T24 and UM‐UC‐3) and a mouse bladder cancer cell line (MB49), the IC_50_ of MP‐12C was significantly lower than those of CDDP and mitomycin C, demonstrating MP‐12C's excellent antitumor proliferative efficacy (Figure 2A and Table 2).

**TABLE 2:** Cell cytotoxicity (IC_50_ in µm) of platinum(IV) prodrugs MP‐12C on bladder cancer cells for 72 h.

Compounds	T24	UM‐UC‐3	MB49
CDDP	0.961 ± 0.040	3.661 ± 0.207	11.371 ± 2.127
Mitomycin C	0.123 ± 0.002	0.165 ± 0.007	3.468 ± 0.845
MP‐12C	0.005 ± 0.000	0.030 ± 0.007	0.260 ± 0.044

T24 bladder cancer cells were categorized into three groups for pharmacological intervention: oxaliplatin, CDDP, and MP‐12C treatments. The Inductively Coupled Plasma Mass Spectrometry (ICP‐MS) analysis results indicated a slight reduction in platinum accumulation within bladder cancer cells in the oxoplatin treatment group; however, after treatment with MP‐12C, which was synthesized by introducing MT and a dodecyl chain at the axial position of oxoplatin, platinum accumulation in bladder cancer cells reached 29.8 times that of the CDDP treatment group (Figure 2B). To further elucidate the cellular absorption capacity of MP‐12C, partition coefficient (log P) values were determined by the shake‐flask method in octanol and water and calculated with Molinspiration miLogP [24]. The log *p* values of the experiment and calculations are listed in Table 3. The log *p* value of MP‐12C was 0.34 ± 0.13 between 0 and 3, which is favorable for cell uptake [25], while that of CDDP was ‐1.22 ± 0.12, suggesting that the high platinum cellular uptake of MP‐12C was consistent with the high lipophilicity. To verify the intracellular activation behavior, the reductive conversion of MP‐12C was investigated in tumor cells. After treatment with 100 µm of MP‐12C for 4 h, cellular extracts were analyzed by HPLC. As shown in Figure S57, a characteristic peak corresponding to the MT derivative (compound **2**, retention time = 13.73 min) was clearly detected, indicating efficient intracellular reduction and release of the axial ligand. These findings confirm that MP‐12C functions as a reduction‐responsive Pt(IV) prodrugs capable of releasing bioactive MT derivatives under intracellular conditions.

**TABLE 3:** Experimental and calculated log *p* value of the compounds.

Compounds	Cal. log P	Exp. log P
CDDP	−4.86	−1.22 ± 0.12
MT	1.45	0.30 ± 0.01
11 (MP‐12C)	1.77	0.34 ± 0.13

The results of the plate colony formation assay indicated that, in comparison to the control, CDDP, and oxoplatin groups, the colony number in the MP‐12C group was significantly reduced, and the colony volume was considerably reduced, implying that MP‐12C's antiproliferative efficacy on T24 cells was significantly superior to that of CDDP and oxoplatin (Figure 2C,E). Subsequently, an EdU proliferation assay was conducted to assess whether MP‐12C's anti‐bladder cancer cell proliferative inhibitory effect was stronger than that of CDDP. The treatment conditions for each cell group were categorized into three intervention groups: control, CDDP, and MP‐12C. These experimental findings were consistent with the conclusions from the plate colony formation assay, demonstrating that MP‐12C's antiproliferative effect on T24 cells was markedly superior to that of CDDP (Figure 2D,F). The extent of apoptosis induction by CDDP and MP‐12C in T24 bladder cancer cells was assessed using apoptosis tests. The findings indicated that, compared with the CDDP group, MP‐12C induced more pronounced apoptosis in T24 cells (Figure 2G,H). Additionally, immunofluorescence staining was performed for the DNA damage marker phosphorylated histone H2AX (γ‐H2AX, Ser139‐phosphorylated histone H2AX) to assess the impact of the medications on DNA damage in T24 bladder cancer cells. The findings indicated that MP‐12C elicited more pronounced DNA damage than CDDP (Figure 2I).

T24 bladder cancer cells were constantly generated and cultivated by progressively increasing the CDDP concentration, ultimately resulting in the successful establishment of CDDP‐resistant T24 (T24/DDP) cells. The CCK8 experiment results indicated that the IC_50_ of CDDP for the parental T24 cells was (0.961 ± 0.040) µm, whereas for the induced T24/DDP cells, it rose to (6.365 ± 0.191) µm, yielding a resistance index of 6.6 (Figure 2J). Notably, MP‐12C maintained robust proliferative inhibitory activity against T24/DDP cells, with an IC_50_ of merely (0.008 ± 0.002) µm, exhibiting no significant reduction in drug efficacy despite the emergence of CDDP resistance in the cells. The IC_50_ of MP‐12C in T24/DDP cells was only 1.6‐fold higher than that in parental T24 cells, whereas CDDP showed a 6.6‐fold increase, indicating that MP‐12C effectively overcame CDDP resistance (Figure 2K).

### 2.5 In Vivo Antitumor Efficacy of MP‐12C When Administered Intravenously in a Cell‐Derived Xenograft (CDX) Model of Bladder Cancer

**FIGURE 3:**
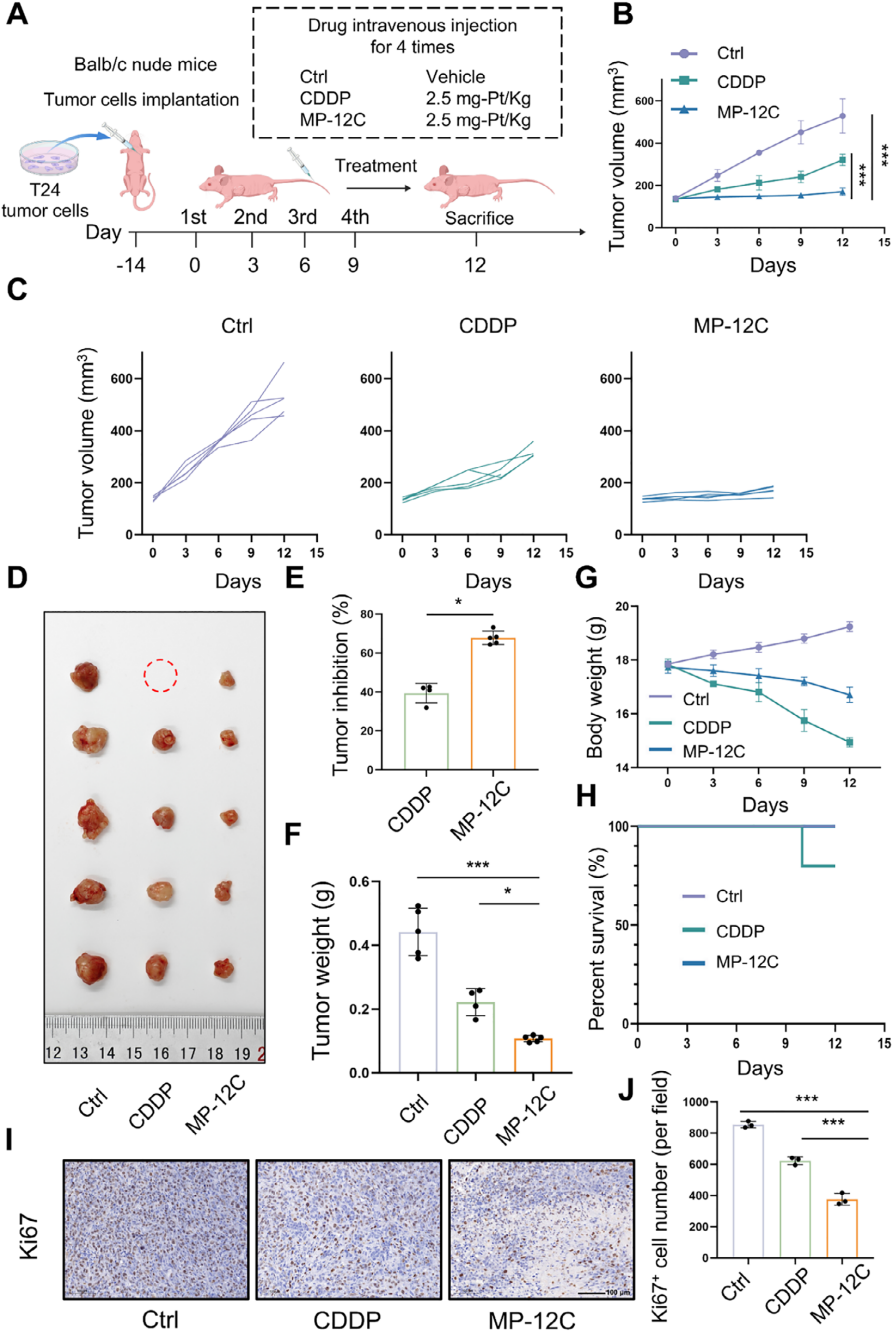
The results of in vivo experiments on a cell‐derived xenograft (CDX) model of T24 tumors (various drugs used for intravenous injection). (A) Pharmacodynamic experimental flowchart (created with BioGDP.com, agreement number: GDP2026NFHW33). (B) Growth curves of tumor volume for each group (n = 5). (C) The changes in tumor volume for each mouse within each group. (D) Macroscopic examination of tumors. (E) Rate of tumor inhibition (for CDDP group: n = 4; for MP‐12C group: n = 5). (F) Tumor mass (for Ctrl group: n = 5; for CDDP group: n = 4; for MP‐12C group: n = 5). (G) Weight variation curves in Balb/c nude mice. (H) Survival curve. (I) Ki67 immunohistochemical labeling was employed to assess tumor growth in the subcutaneous tumor tissue of Balb/c nude mice (n = 3). (J) Statistical analysis of the Ki67‐positive cell count. Data are presented as mean ± SD. Statistical analysis was performed using one‐way ANOVA followed by Tukey's post‐hoc test (B, F, J). Statistical analysis was performed using a two‐sided Mann‐Whitney U test (E). ^*^ *p* < 0.05, ^**^ *p* < 0.01, ^***^ *p* < 0.001; ns, not significant.

To further validate the in vivo efficacy of MP‐12C as an intravenous therapeutic agent, a subcutaneous tumor model with T24 cell xenografts was established in BALB/c nude mice. Figure 3A illustrates a flowchart. Fourteen days post‐modeling, mice with tumor volumes between 150 and 200 mm^3^ were randomly assigned to three groups of five mice each. The precise classification and dosage schedules were as follows: Control, CDDP (2.5 mg platinum/kg), and MP‐12C (2.5 mg platinum/kg) groups. The drug was administered every three days for a total of four administrations. Tumor volume and body weight changes in nude mice were systematically monitored and documented throughout the dosing period. Figure 3B–D illustrates that, compared with the control and CDDP groups, the tumor volume in the nude mice of the MP‐12C group was significantly reduced. The tumor inhibition rate in the CDDP group was 39.4%, whereas that in the MP‐12C group was 67.8% (Figure 3E). Concurrently, compared with the control and CDDP groups, tumor weight in nude mice in the MP‐12C group was significantly reduced (Figure 3F). Weight loss in nude mice in the CDDP group was markedly greater than that in the MP‐12C group (Figure 3G). During the administration period, one mouse in the CDDP group died on the 10th day, whereas no fatalities were observed in the control or MP‐12C groups (Figure 3H). Furthermore, Ki67 immunohistochemical (IHC) assay results indicated that, compared with the control and CDDP groups, MP‐12C treatment significantly reduced the proliferative activity of tumor cells (Figure 3I,J).

The Hematoxylin‐Eosin (HE) staining of the principal organs (including the heart, liver, spleen, lung, and kidney) of nude mice in both the control and CDDP groups (Figure S51) demonstrated that MP‐12C exhibited an improved safety profile relative to CDDP.

### 2.6 In Vivo Antitumor Efficacy of the MP‐12C When Administered Intravenously in an In Situ Model of Bladder Cancer

**FIGURE 4:**
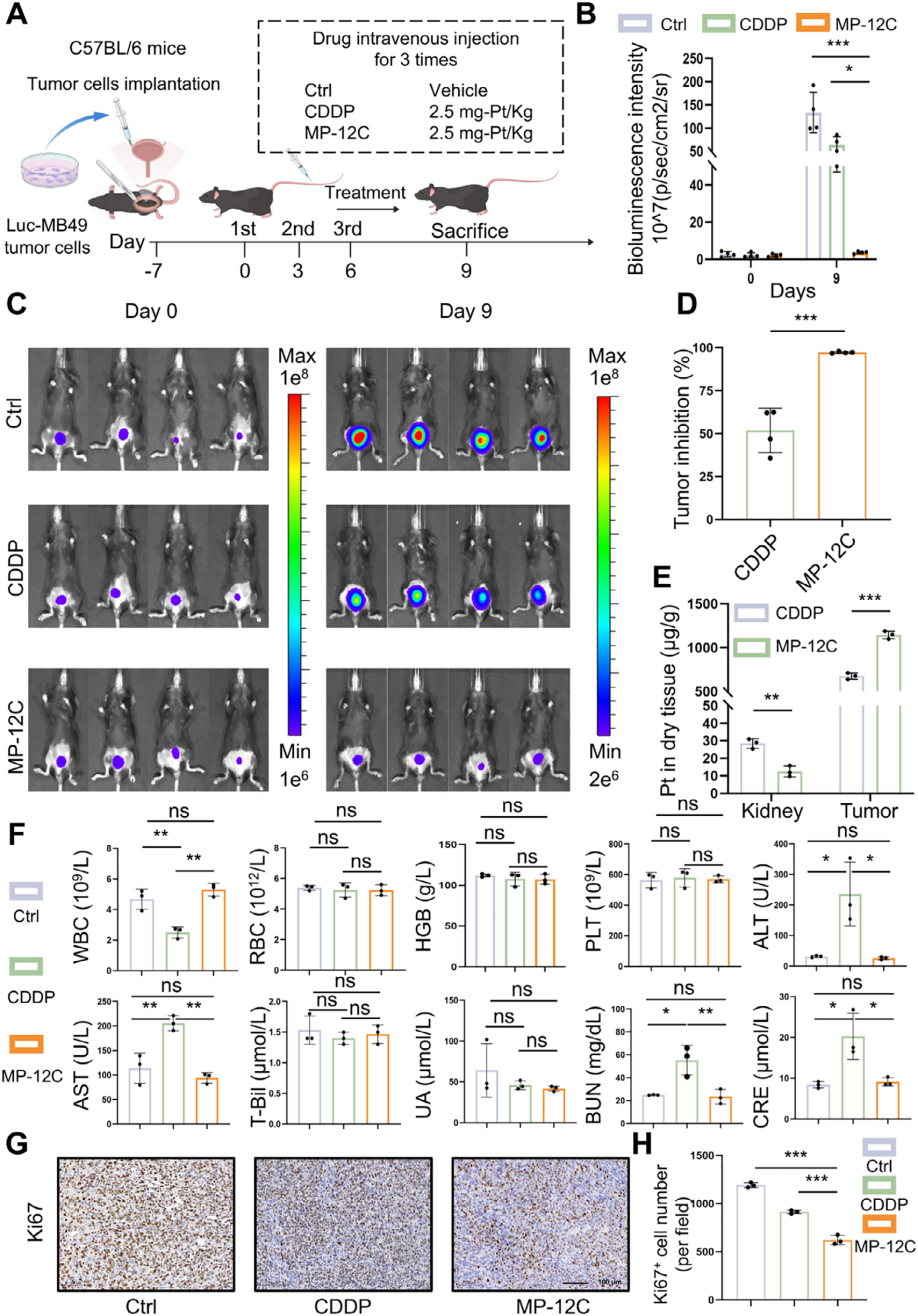
The results of in vivo experiments on an in situ model of MB49 tumors (various drugs used for intravenous injection). (A) Pharmacodynamic experimental flowchart (created with BioGDP.com, agreement number: GDP20265FHW42). (B) ROI value for imaging C57BL/6 mice (n = 4). (C) Imaging of C57BL/6 mice. (D) Tumor suppression rate (n = 4). (E) ICP‐MS analysis of platinum accumulation in the kidney and tumor tissues of mice across all groups (n = 3). (F) The outcomes of routine blood examinations and biochemical analyses for each mouse following various intravenous medication treatments (n = 3). (G) Ki‐67 immunohistochemical labeling was used to assess tumor proliferation in situ within the tumor tissue of C57/BL6 mice. (H) Statistical study of Ki67‐positive cell count (n = 3). Data are presented as mean ± SD. Statistical analysis was performed using one‐way ANOVA followed by Tukey's post‐hoc test (B, F (except for T‐Bil),H). Statistical analysis was performed using Kruskal–Wallis H test with Dunn's post‐hoc correction (F (T‐Bil)). Statistical analysis was performed using two‐sided Student's *t*‐test (D,E). ^*^ *p* < 0.05, ^**^ *p* < 0.01, ^***^ *p* < 0.001; ns, not significant.

To further examine the in vivo antitumor efficacy of MP‐12C as an intravenous therapeutic agent in a bladder cancer in situ model with preserved immunity, a bladder cancer in situ xenograft model utilizing C57BL/6 mice implanted with Luc‐MB49 cells was established. Figure 4A illustrates the flowchart. Seven days post‐modeling, mice with comparable region‐of‐interest (ROI) values were selected and randomly assigned to three groups of four mice each. Statistical analysis indicated no significant differences in ROI values across the groups. The experimental groups and dosing regimen were as follows: control, CDDP (2.5 mg platinum/kg), and MP‐12C (2.5 mg platinum/kg) groups, administered once every three days for a total of three administrations. Compared to the control and CDDP groups, the ROI value (Figure 4B,C) and the number of Ki67‐positive cells in the tumor tissue (Figure 4G,H) in the MP‐12C group were significantly reduced. Furthermore, the tumor inhibition rate in the CDDP group was 51.9%, whereas the MP‐12C group exhibited a significantly higher rate of 97.3% (Figure 4D). ICP‐MS results indicated that platinum accumulation in the kidneys of the MP‐12C group was markedly lower than that in the CDDP group, whereas platinum accumulation in the tumor was significantly higher than that in the CDDP group, implying that MP‐12C possessed a more effective and less toxic antitumor advantage than CDDP (Figure 4E).

The results of routine blood and biochemical tests showed no statistically significant differences in any detection parameters between the control and MP‐12C groups of C57BL/6 mice. In contrast, the white blood cell (WBC) count in the CDDP group was significantly reduced, while alanine transaminase (ALT), aspartate aminotransferase (AST), blood urea nitrogen (BUN), and creatinine (CRE) levels were significantly elevated (Figure 4F). These results indicated that CDDP decreased the WBC count and caused liver and kidney function damage in mice, whereas no obvious abnormalities were observed in the MP‐12C group after intervention, further confirming the low‐toxicity in vivo drug characteristics of MP‐12C.

### 2.7 In Vivo Antitumor Efficacy of the MP‐12C When Administered Intravesically in an In Situ Model of Bladder Cancer

**FIGURE 5:**
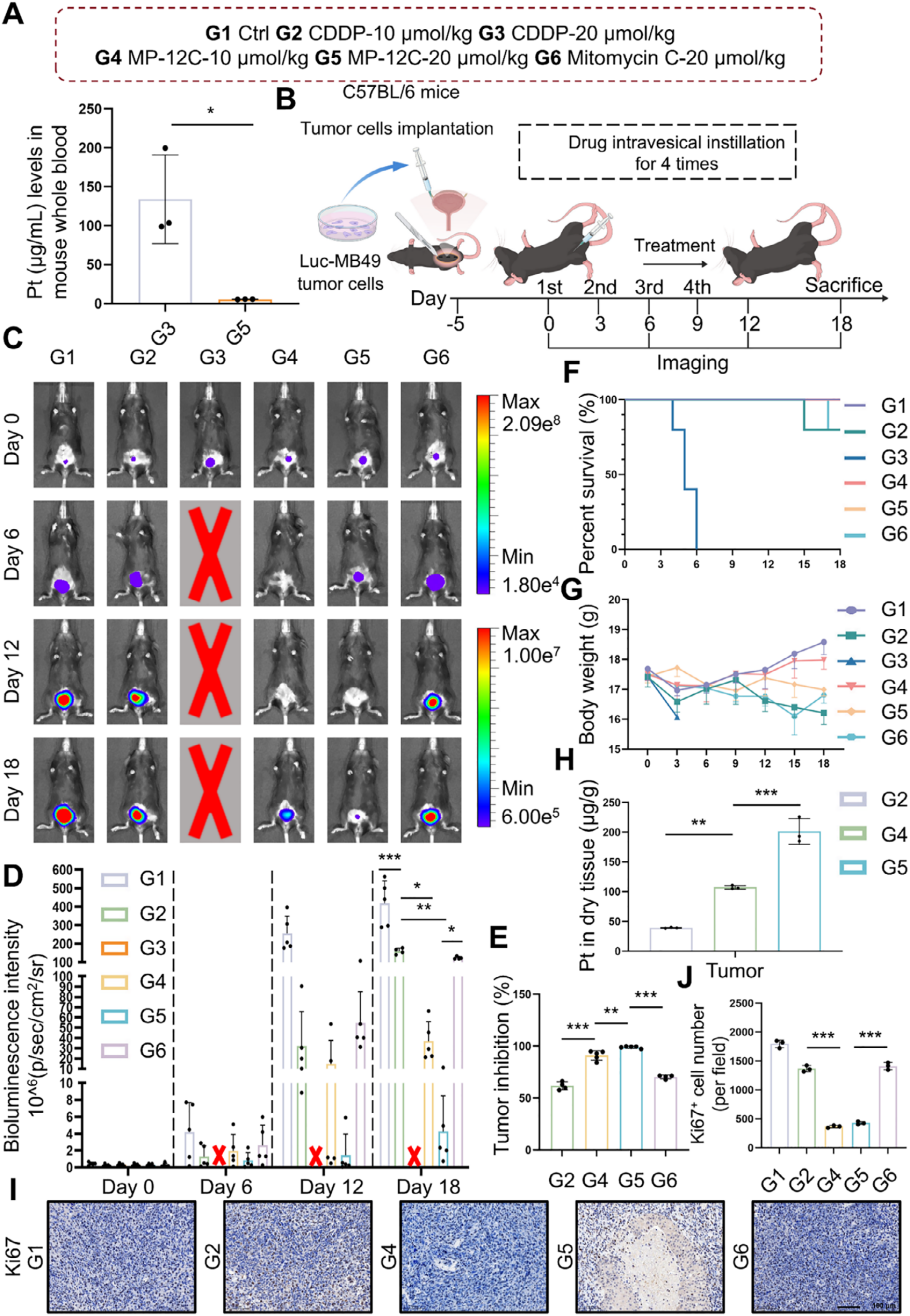
The results of in vivo experiments on an in situ model of MB49 tumors (various drugs used for bladder instillation). (A) Detection of platinum exposure in whole blood of normal C57BL/6 mice via ICP‐MS following a 1‐h bladder instillation of CDDP or MP‐12C (n = 3). (B) Pharmacodynamic experimental flowchart (created with BioGDP.com, agreement number: GDP2026PFHABT). (C) Imaging of C57BL/6 mice. (D) ROI value for imaging C57BL/6 mice (for G1: n = 5; for G2: n = 4; for G3: n = 0; for G4: n = 5; for G5: n = 5; for G6: n = 4). (E) Tumor suppression rate (for G1: n = 5; for G2: n = 4; for G3: n = 0; for G4: n = 5; for G5: n = 5; for G6: n = 4). (F) Survival curve. (G) Weight variation curves in C57/BL6 mice. (H) ICP‐MS analysis of platinum accumulation in the tumor tissues of mice across all groups (n = 3). (I) Ki67 immunohistochemistry labeling was employed to assess tumor proliferation in situ inside the tumor tissue of C57/BL6 mice. (J) Statistical study of Ki67‐positive cell count (n = 3). Data are presented as mean ± SD. Statistical analysis was performed using a two‐sided Student's *t*‐test (A). Statistical analysis was performed using one‐way ANOVA followed by Tukey's post‐hoc test (D, E, H, J). ^*^ *p* < 0.05, ^**^ *p* < 0.01, ^***^ *p* < 0.001; ns, not significant.

Mice were initially administered an intravesical instillation of CDDP and MP‐12C at 20 µmol/kg each. One hour after delivery, ICP‐MS was used to quantify platinum concentration in whole blood from the mice. The results demonstrated that the platinum content in whole blood after the intravesical infusion of MP‐12C was significantly lower than that of CDDP, suggesting that MP‐12C's in vivo toxicity was substantially lower than that of CDDP when administered intravesically (Figure 5A).

The antitumor efficacy and in vivo toxicity of MP‐12C, CDDP, and mitomycin C were further evaluated in the C57BL/6 mice Luc‐MB49 bladder cancer orthotopic tumor model. Figure 5B shows the flowchart. Five days after modeling, mice with similar ROI values were randomly assigned to six groups of five mice each. Statistical analysis revealed no significant differences in ROI values among the groups. The experimental categories and dosage regimen were delineated as follows: control, low‐dose CDDP (10 µmol/kg), high‐dose CDDP (20 µmol/kg), low‐dose MP‐12C (10 µmol/kg), high‐dose MP‐12C (20 µmol/kg), and mitomycin C (20 µmol/kg) groups, administered once every three days for a total of four doses. As shown in Figure 5C,D, the tumor ROI value in the low‐dose MP‐12C group was significantly reduced relative to the control, low‐dose CDDP, and mitomycin C groups, and the ROI value in the high‐dose MP‐12C group decreased even further. Quantitative analysis of the ROI values revealed that the tumor inhibition rates for the low‐dose CDDP and mitomycin C groups were 61.7% and 70.4%, respectively, whereas the tumor inhibition rates for the low‐dose and high‐dose MP‐12C groups were 91.1% and 99.0%, respectively (Figure 5E). During the drug administration phase, all mice in the high‐dose CDDP group died after two doses; at the end of the experiment, one mouse died in both the low‐dose CDDP group and the mitomycin C group, while no deaths were recorded in the control, low‐dose MP‐12C, and high‐dose MP‐12C groups (Figure 5F). The weight variations of mice in the low‐dose MP‐12C and control groups were similar; however, the weight of mice in the high‐dose MP‐12C group showed a slight decrease, which was significantly smaller than that observed in the low‐dose CDDP group (Figure 5G).

The ICP‐MS results demonstrated that platinum accumulation in the tumors of the MP‐12C group was markedly greater than that in the CDDP group, thus reinforcing the assertion that MP‐12C has superior antitumor efficacy relative to CDDP (Figure 5H). Ki67 IHC analysis of tumor tissues indicated that, compared with the control, low‐dose CDDP, and mitomycin C groups, MP‐12C intervention more effectively suppressed tumor cell proliferative activity (Figure 5I,J). The results of routine blood tests, biochemical tests (Figure S52), and main organ HE staining (Figure S53) revealed that WBC and red blood cell (RBC) counts in the low‐dose CDDP and mitomycin C groups exhibited varying degrees of reduction, while hemoglobin (HGB) levels in the mitomycin C group significantly declined; concurrently, both the low‐dose CDDP and mitomycin C groups demonstrated varying degrees of liver and kidney function impairment. In summary, MP‐12C demonstrated markedly superior antitumor activity and significantly reduced systemic toxicity compared to CDDP and mitomycin C as a therapy for bladder cancer via intravesical instillation.

### 2.8 In Vivo Antitumor Efficacy of the MP‐12C When Administered Intravesically in the N‑butyl‑N‑(4‑Hydroxybutyl)Nitrosamine (BBN)‐Induced Spontaneous Model of Bladder Cancer

**FIGURE 6:**
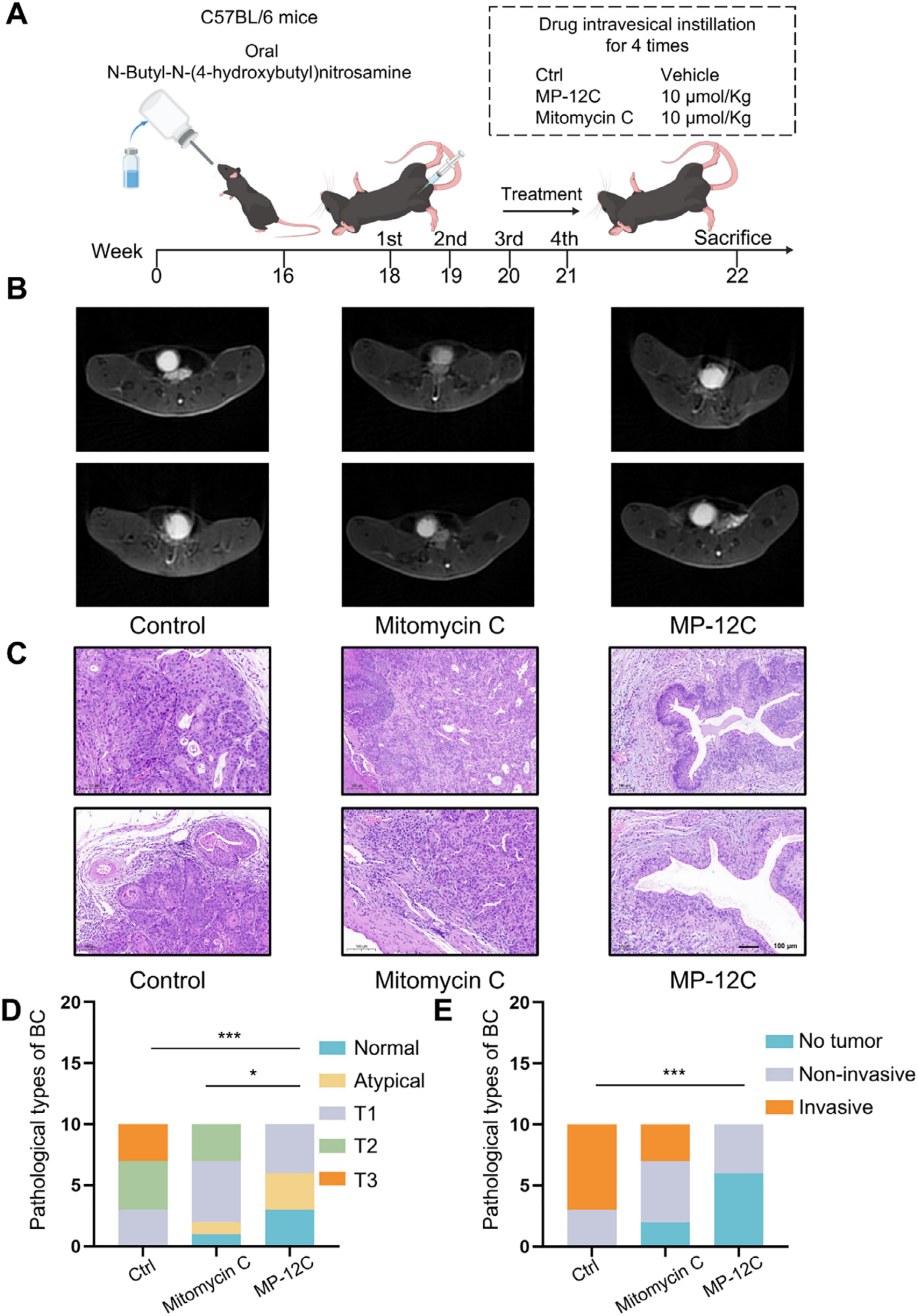
The results of in vivo experiments on a spontaneous bladder cancer model induced by BBN (various drugs used for bladder instillation). (A) Pharmacodynamic experimental flowchart (created with BioGDP.com, agreement number: GDP2026OTJA23). (B) Representative MRI images. (C) Representative histological examination of bladder carcinoma. (D) Statistical examination of the pathological T stage of bladder cancer in mice across each group (n = 10). (E) Statistical examination of the progressive stages of bladder cancer in mice from each group (n = 10). Data are presented as mean ± SD. Statistical analysis was performed using a two‐sided Mann‐Whitney U test (D,E). ^*^ *p* < 0.05, ^**^ *p* < 0.01, ^***^ *p* < 0.001; ns, not significant.

Initially, C57BL/6 mice were administered drinking water infused with 0.05% BBN for 4 months to establish a spontaneous bladder cancer model. At the 18th week, hematuria was assessed using urine test strips in the mice, and magnetic resonance imaging (MRI) was employed to identify animals that had developed spontaneous bladder cancer, which were subsequently included in the pharmacodynamic investigations (Figure 6A,B). Following screening, the mice underwent treatment via intravesical instillation weekly for four weeks, after which the treatment was halted for one week before euthanization. Bladder tissues were harvested and processed for HE staining (Figure 6C). Histological analysis indicated that, compared with the mitomycin C and control groups, the bladder tumor T stage in the MP‐12C group was generally lower (Figure 6D), and the incidence of MIBC was greatly reduced (Figure 6E). Given that the BBN‐induced spontaneous model closely recapitulates the stepwise progression from NMIBC to MIBC in humans, this result suggests that MP‐12C has the potential to block disease stage advancement in bladder cancer and has good application prospects for NMIBC.

### 2.9 Transcriptome Sequencing for CDDP Chemotherapy Sensitization Mechanisms of MT

To elucidate the potential molecular mechanism underlying MT's chemosensitizing effect on CDDP, the human bladder cancer cell line T24 was divided into three treatment groups: control, CDDP, and MP‐12C. Cells from each group were harvested, and total RNA was extracted for transcriptome sequencing analysis.

**FIGURE 7:**
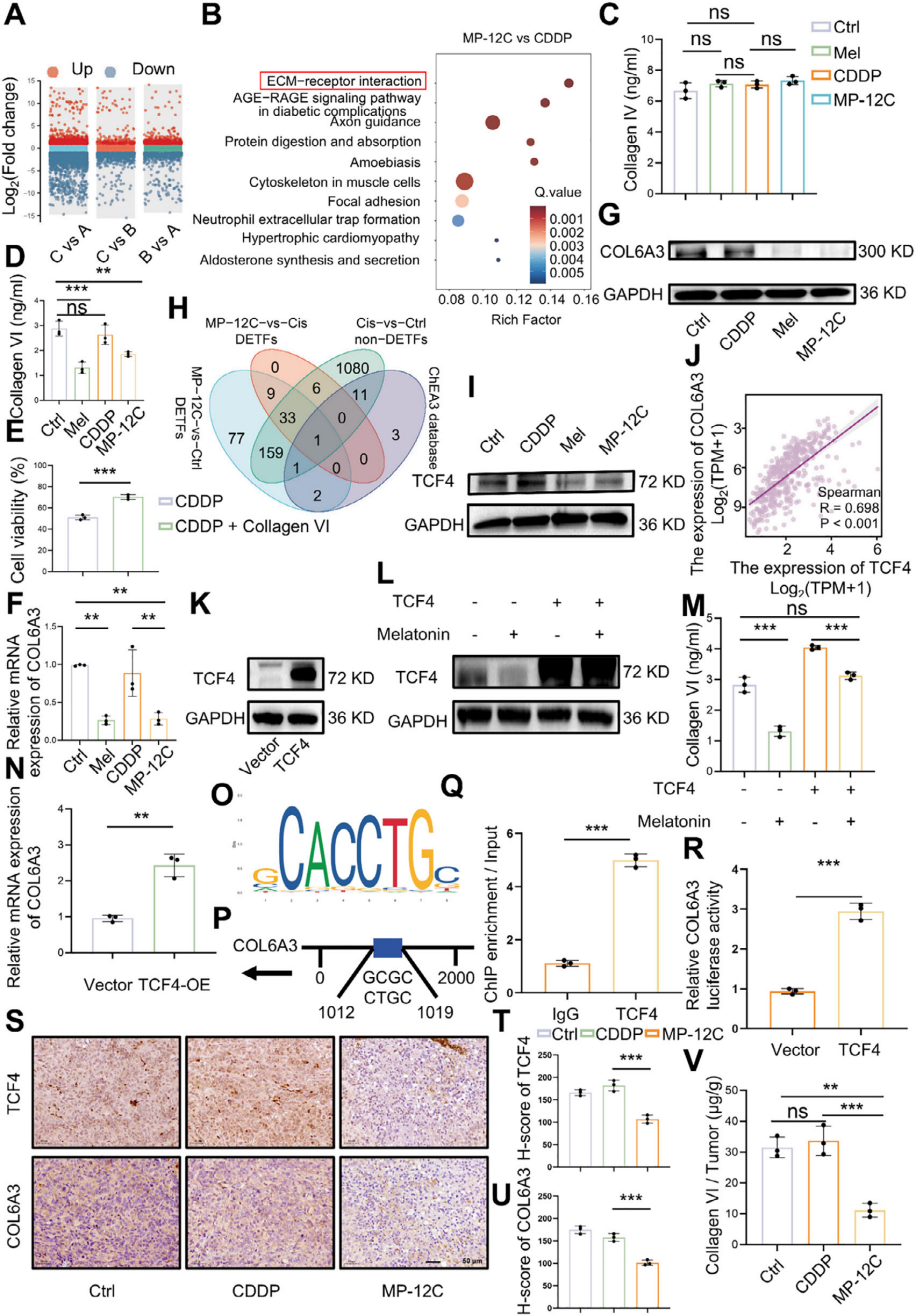
The mechanism by which melatonin can enhance CDDP sensitivity. (A) Transcriptome sequencing of T24 cells treated with control (Group A), CDDP (Group B), and MP‐12C (Group C). Volcanic map of DEGs compared pairwise between groups. (B) Enrichment analysis of DEGs between the MP‐12C group and the CDDP group using the KEGG database. (C) ELISA was used to detect the secretion level of type IV collagen protein in T24 cells from different treatment groups (n = 3). (D) ELISA was used to detect the secretion level of type VI collagen in T24 cells from different treatment groups (n = 3). (E) CCK‐8 Cell Viability Assay was used to detect the effect of type VI collagen on the viability of T24 cells after CDDP treatment (n = 3). (F) *COL6A3* mRNA expression in T24 cells from different treatment groups (n = 3). (G) COL6A3 protein expression in T24 cells from different treatment groups. (H) Venn diagram: By analyzing and combining the transcriptome differential dataset with the CHEA3 database, the upstream candidate transcription factor TCF4 of *COL6A3* was identified and selected. (I) TCF4 protein expression in T24 cells from each group. (J) The correlation between the transcription levels of *TCF4* and *COL6A3* in the transcriptome data of the TCGA‐BLCA cohort. (K) The stable overexpression efficiency of TCF4 in T24 cells using GAPDH as the internal control. (L) In the rescue experiment, the expression levels of TCF4 protein in control and TCF4‐overexpressing T24 cells were assessed after treatment with DMSO or melatonin, using GAPDH as an internal control. (M) In the rescue experiment, ELISA was used to detect the secretion level of type VI collagen in T24 cells from different treatment groups (n = 3). (N) The effect of TCF4 overexpression on the expression level of *COL6A3* mRNA in T24 cells (n = 3). (O) The core binding motifs of the TCF4 protein were predicted by the JASPAR database. (P) COL6A3 promoter region binding site predicted by the JASPAR database. (Q) The specific binding of the TCF4 protein to the COL6A3 promoter was verified through ChIP experiments, with IgG serving as the negative control (n = 3). (R) The direct activation effect of the TCF4 protein on the transcriptional activity of the COL6A3 promoter was verified through a dual‐luciferase reporter gene assay (n = 3). (S) TCF4 and COL6A3 immunohistochemical staining were used to detect tumor proliferation in subcutaneous tumor tissue of Balb/c nude mice. (T) Statistical analysis of the H‐score of IHC‐stained COL6A3 (n = 3). (U) Statistical analysis of the H‐score of IHC‐stained TCF4 (n = 3). (V) ELISA was used to detect the collagen VI content in subcutaneous tumors of Balb/c nude mice (n = 3). Statistical analysis was performed using one‐way ANOVA followed by Tukey's post‐hoc test (C, D, F, M, T–V). Statistical analysis was performed using a two‐sided Student's *t*‐test (E, N, Q, R). ^*^ *p* < 0.05, ^**^ *p* < 0.01, ^***^ *p* < 0.001; ns, not significant.

The sequencing results indicated that, relative to the control group, the CDDP group exhibited 1073 differentially expressed genes (DEGs), comprising 458 upregulated and 615 downregulated genes. Compared to the control group, the MP‐12C group revealed 3079 DEGs, including 1142 up‐regulated and 1937 down‐regulated genes. Furthermore, compared with the CDDP group, the MP‐12C group exhibited 853 DEGs, comprising 177 upregulated and 676 downregulated genes (Figure 7A). Gene Ontology (GO) (Figure S54A) and Kyoto Encyclopedia of Genes and Genomes (KEGG) enrichment analyses (Figure 7B) were performed on DEGs between the MP‐12C and CDDP groups. The findings indicated that MP‐12C therapy markedly modulated the biological processes and signaling pathways associated with the ECM. The GO enrichment analysis revealed marked enrichment of fundamental components, including collagen‐containing extracellular matrix and extracellular matrix structural constituents. The KEGG analysis revealed a marked enhancement in the ECM‐receptor interaction pathway. Nonetheless, following the execution of GO (Figure S54B) and KEGG (Figure S54C) analyses on the DEGs between the CDDP and control groups, no notable enrichment of extracellular matrix‐related items and pathways was detected.

Figure S55 illustrates the Gene Set Enrichment Analysis (GSEA) performed on DEGs between the MP‐12C and CDDP groups to elucidate the primary functional pathways of MT and MP‐12C in modulating CDDP chemosensitivity. The results demonstrated significant enrichment at the KEGG pathway level in the ECM‐receptor interaction pathway. At the GO enrichment level, it exhibited substantial enrichment in ECM‐related entries across three ontologies: molecular function (MF), cellular component (CC), and biological process (BP), outlined as follows: MF: ECM structural constituent, integrin binding, ECM structural constituent conferring tensile strength, ECM binding, collagen binding; CC: collagen containing ECM, collagen trimer, ECM; BP: cell matrix adhesion, ECM organization, collagen fibril organization, positive regulation of cell matrix adhesion, cell adhesion mediated by integrin.

### 2.10 MT Facilitates Sensitization to CDDP Chemotherapy by Regulating Collagen VI Secretion in Bladder Cancer Cells

Figure S56A illustrates that by intersecting the genes associated with the ECM‐receptor interaction pathway between the MP‐12C and control groups with those between the MP‐12C and CDDP groups, and then intersecting these results with the genes showing no differences between the CDDP and control groups, a total of 13 distinct ECM‐receptor interaction pathway genes were identified. Six collagen‐encoding genes were identified, specifically collagen IV genes (*COL4A1*, *COL4A2*, *COL4A3*, *COL4A4*, *COL4A5*) and the collagen VI gene (*COL6A3*) (Figure S56B).

Enzyme‐Linked Immunosorbent Assay‌ (ELISA) was employed to assess the secretion levels of collagen IV and VI in T24 cells following various pharmacological treatments to ascertain whether the differential expression of collagen genes identified through transcriptome sequencing corresponded to changes in collagen secretion levels. As shown in Figure 7C,D, the CDDP treatment did not significantly alter the basal secretion levels of collagen IV and VI in T24 cells. However, MT and MP‐12C treatment did not significantly affect collagen IV secretion, but significantly modulated collagen VI secretion in T24 cells.

The CCK‐8 assay was used to elucidate the role of collagen VI in CDDP chemosensitivity by assessing the proliferative activity and drug responsiveness of T24 cells. The findings indicated that collagen VI significantly reduced the chemosensitivity of T24 cells to CDDP, thereby counteracting CDDP's proliferation‐inhibitory action and attenuating its antitumor efficacy (Figure 7E). To determine whether collagen VI is a necessary mediator of MP‐12C‐induced chemosensitization, exogenous collagen VI rescue experiments were performed. The cell inhibition rate was 20.3% for MP‐12C alone, which decreased to 13.3% upon collagen VI supplementation, indicating that collagen VI reversed approximately 7.0% of MP‐12C ‐induced cytotoxicity; the inhibition rate was 44.8% for CDDP alone, which decreased to 24.8% upon collagen VI supplementation, indicating that collagen VI reversed approximately 20.0% of CDDP ‐induced cytotoxicity; and the inhibition rate was 85.2% for the MP‐12C + CDDP combination, which decreased to 43.9% upon collagen VI supplementation, indicating that collagen VI reversed approximately 41.3% of the combination‐induced cytotoxicity. These results demonstrate that collagen VI not only partially reverses the anti‐tumor effects of MP‐12C and CDDP as single agents, but more importantly, the magnitude of reversal for the combination treatment (41.3%) is substantially greater than the sum of reversals for each individual drug (7.0% + 20.0% = 27.0%). This differential effect suggests that collagen VI specifically attenuates the chemosensitizing effect of MP‐12C on CDDP (Figure S58).

*COL6A3*, the gene encoding collagen VI, was the sole gene within the collagen VI family and exhibited significant differential expression in the ECM‐receptor interaction pathway when comparing the MP‐12C group to both the control and CDDP groups, with no significant differences observed between the two groups (Figure S56B). To elucidate the primary molecular targets of MT and MP‐12C in modulating collagen VI secretion in T24 cells, and to validate the transcriptome sequencing findings, ‌Quantitative Reverse Transcription Polymerase Chain Reaction (qRT‐PCR) and Western Blotting assays‌ were employed to assess the mRNA and protein levels of *COL6A3* in T24 cells following various drug treatments. The results showed that CDDP treatment had no significant regulatory effect on *COL6A3* mRNA and protein expression levels in T24 cells, whereas MT and MP‐12C treatments significantly downregulated *COL6A3* mRNA and protein expression levels in T24 cells. This outcome is consistent with the transcriptome sequencing findings (Figure 7F,G).

### 2.11 MT Modulates the Release of Collagen VI via the Transcription Factor 4 (*TCF4*)/*COL6A3* Pathway, Thereby Enhancing the Sensitivity of Bladder Cancer Cells to CDDP Treatment

According to the previously mentioned research findings, *COL6A3* is a crucial target for the sensitization effects of MT and MP‐12C in CDDP chemotherapy. Transcription factors are fundamental regulators of gene expression and have been shown to play significant roles in the malignant evolution of bladder cancer and in modulating chemotherapy sensitivity. In this study, the fundamental screening criterion of “specifically regulated by MT/MP‐12C (|log_2_FC| > 1, *q*‐value < 0.05) and unaffected by CDDP (*q*‐value > 0.05)” was employed to analyze transcriptome sequencing data. Initially, the intersection of significantly differentially expressed transcription factors was identified between the MP‐12C and control groups and between the MP‐12C and CDDP groups. Subsequently, secondary screening was performed using transcription factors that did not exhibit significant differential expression between the CDDP and control groups, thereby yielding candidate transcription factors that adhered to the screening criterion. Furthermore, the intersection of this candidate set with the potential upstream transcriptional regulatory factor set of COL6A3, as predicted by the ChEA3 database within the TF Target Finder database. In the end, *TCF4* was the only candidate transcription factor obtained after screening (Figure 7H). CDDP did not affect TCF4 protein expression, whereas both MT and MP‐12C reduced it (Figure 7I).

Analysis of transcriptome data from The Cancer Genome Atlas (TCGA) revealed a correlation coefficient of 0.698 between the mRNA expression levels of *TCF4* and *COL6A3*, indicating a strong association (Figure 7J). After a T24 cell line with sustained overexpression of *TCF4* was established (Figure 7K), the reversal experiment showed that MT reduced TCF4 protein expression in T24 cells. However, the overexpression of *TCF4* significantly restored the effects of MT on the protein expression level of TCF4 in T24 cells (Figure 7L) and the secretion level of collagen VI (Figure 7M). This confirmed that MT reduced collagen VI secretion in bladder cancer cells by downregulating TCF4 expression, thereby enhancing CDDP sensitivity in T24 bladder cancer cells.

In this study, the regulatory role of the TCF4 protein in *COL6A3* and its molecular mechanism were elucidated, revealing that TCF4 significantly upregulated *COL6A3* mRNA levels (Figure 7N). Additionally, the JASPAR database predicted the core binding motif of the TCF4 protein, identifying a high‐confidence binding site within the *COL6A3* promoter region (Figure 7O,P). A Chromatin Immunoprecipitation assay (ChIP) confirmed that TCF4 directly bound to the *COL6A3* promoter, with 4.82‐fold enrichment compared to the IgG control (Figure 7Q). Dual‐luciferase reporter gene assays confirmed that the TCF4 protein directly bound to the *COL6A3* promoter, thereby positively regulating *COL6A3* transcription. Specifically, the enrichment level in the *TCF4* overexpression group was 2.9 times higher (Figure 7R).

The T24 cell subcutaneous xenograft tumor model was utilized to validate the regulatory influence of MP‐12C on the *TCF4*/*COL6A3* axis in vivo based on the established molecular mechanism by which “MT inhibits the transcriptional expression of the target gene *COL6A3* by downregulating the transcription factor *TCF4*, thereby facilitating CDDP chemosensitization,” as evidenced by prior in vitro cell experiments. The expression levels of the target genes in the tumor tissues of each group of mice were assessed by IHC staining. The findings indicated that, in comparison to the control and CDDP groups, the expression levels of TCF4 and COL6A3 proteins in the tumor tissues of mice treated with MP‐12C were markedly downregulated, aligning perfectly with the outcomes of the in vitro cell experiments (Figure 7S–U); additionally, and that the concentration of collagen VI in the tumor tissues of the MP‐12C treatment group was significantly reduced (Figure 7V). The in vivo experimental data further validated the effects of Melatonin on the *TCF4*/*COL6A3* signaling axis and collagen VI.

### 2.12 MP‐12C Causes Immunogenic Cell Death and Stabilizes the Activation of the Cgas‐STING Pathway in Bladder Cancer

**FIGURE 8:**
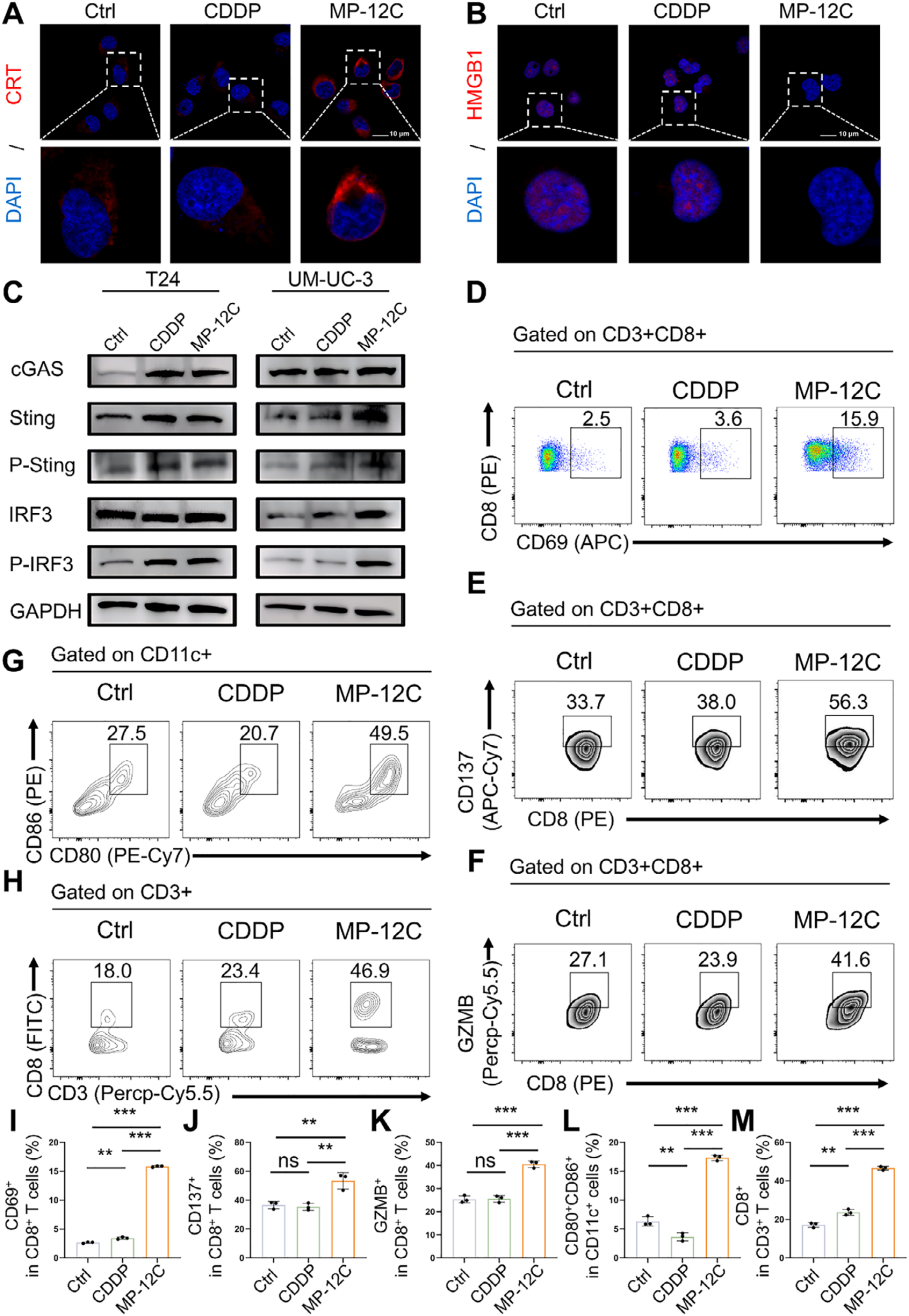
MP‐12C reshapes the immune microenvironment of bladder cancer. (A) MP‐12C promotes the exposure of CRT on the membrane surface of T24 cells. (B) MP‐12C produces a reduction in *HMGB1* nuclear expression in T24 cells. (C) MP‐12C consistently activates the cGAS‐STING pathway in T24 and UM‐UC‐3 cells. (D) Flow cytometry analysis of CD69 expression, an early activation marker, on CD8^+^ T cells in an in vitro study. (E) Flow cytometry analysis of CD137 expression, a hallmark of chronic activation, on CD8^+^ T cells in in vitro studies. (F) Flow cytometry analysis of GZMB effector molecule expression in CD8^+^ T cells during in vitro experiments. (G) Flow cytometry assessment of the infiltration percentage of CD11c^+^CD80^+^CD86^+^ dendritic cells in tumor tissues. (H) Flow cytometry analysis of the infiltration percentage of CD3^+^CD8^+^ T cells within tumor tissues. (I) Statistical examination of the number of CD69^+^CD8^+^ T cells across several treatment groups in in vitro trials (n = 3). (J) Statistical examination of the number of CD137^+^CD8^+^ T cells across several treatment groups in in vitro trials (n = 3). (K) Statistical examination of the amount of GZMB^+^CD8^+^ T cells across several treatment groups in in vitro experiments (n = 3). (L) Statistical examination of the fraction of CD11c^+^CD80^+^CD86^+^ dendritic cells in various treatment groups in an in vivo experiment (n = 3). (M) Statistical examination of the fraction of CD3^+^CD8^+^ T cells across several treatment groups in an in vivo experiment (n = 3). Statistical analysis was performed using one‐way ANOVA followed by Tukey's post‐hoc test (I–M). ^*^ *p* < 0.05, ^**^ *p* < 0.01, ^***^ *p* < 0.001; ns, not significant.

In this study, the ability of MP‐12C to induce ICD was first confirmed in human bladder cancer T24 cells to assess its remodeling effects on the immune microenvironment of bladder cancer. T24 cells were randomly allocated to three groups: control, CDDP, and MP‐12C. Following treatment with appropriate pharmaceuticals, the expression and location of critical molecules involved in ICD were examined by immunofluorescence staining. The findings indicated that MP‐12C treatment markedly induced ICD in T24 cells, facilitating the release and presentation of damage‐associated molecular patterns (DAMPs). The surface exposure of calreticulin (CRT) on the cell membrane greatly increased (Figure 8A), whereas the nuclear expression of high mobility group box 1 (HMGB1) significantly decreased (Figure 8B). The results indicated that MP‐12C modified the immunogenicity of bladder cancer cells by triggering ICD, thereby establishing a basis for enhancing the antitumor response.

The cGAS‐STING pathway is a fundamental signaling axis that modulates the tumor immune microenvironment. The remodeling mechanism of MP‐12C within the immunological microenvironment of bladder cancer was elucidated by confirming activation of the cGAS‐STING pathway in two bladder cancer cell lines. T24 and UM‐UC‐3 cells were allocated into three groups: control, CDDP treatment, and MP‐12C treatment. Following administration of the respective pharmaceuticals, the total and phosphorylated protein expression levels of critical components in the cGAS‐STING pathway were assessed by Western Blotting. Figure 8C illustrates the results. In T24 cells, both CDDP and MP‐12C treatment markedly increased the protein expression levels of cGAS, STING, P‐STING, and P‐IRF3, indicating their ability to activate the cGAS‐STING pathway. Conversely, in UM‐UC‐3 cells, CDDP treatment did not significantly alter the expression of critical molecules in the cGAS‐STING pathway, whereas MP‐12C treatment significantly increased the protein levels of STING, P‐STING, IRF3, and P‐IRF3 without notably affecting cGAS expression.

According to the above results, MP‐12C successfully activated the cGAS‐STING signaling pathway in both T24 and UM‐UC‐3 bladder cancer cell lines, and the activating effect of CDDP was dependent on the cell line. This disparity indicated that, in contrast to CDDP, MP‐12C engaged the cGAS‐STING pathway in a broader cohort of patients with bladder cancer, thereby more effectively modifying the tumor immune milieu and establishing a basis for augmenting the antitumor response.

### 2.13 MP‐12C Facilitates the Activation and Differentiation of CD8^+^ T Cells In Vitro

This study aimed to elucidate whether MP‐12C could successfully activate adaptive antitumor immune responses, based on data indicating that MP‐12C dramatically induces ICD in T24 cells, releases DAMPs, and concurrently activates the cGAS‐STING pathway. A co‐culture system comprising tumor cell culture supernatants and peripheral blood mononuclear cells (PBMC) was established to assess the regulatory impact of MP‐12C therapy on the early activation of CD8^+^ T cells. T24 cells were randomly allocated into three groups: control, CDDP, and MP‐12C. Following the matching pharmacological intervention, the culture supernatants from each group were collected. Following the co‐culturing of supernatants with PBMC suspensions from healthy individuals for 24 h, the number of cells simultaneously expressing the early activation marker CD69 in CD3^+^CD8^+^ T cells was assessed by flow cytometry (gating strategy: focused on CD3^+^CD8^+^ T cells). The results indicated that, compared to the control group, the proportion of CD69‐positive CD3^+^CD8^+^ T cells in the CDDP group showed a modest, statistically significant increase; however, compared with both the control and CDDP groups, the MP‐12C group exhibited a marked, statistically significant upregulation of CD69‐positive CD3^+^CD8^+^ T cells. This outcome confirmed that MP‐12C treatment markedly enhanced the early activation of CD8^+^ T lymphocytes (Figure 8D,I).

Building on the previously reported finding that MP‐12C markedly enhanced the early activation of CD8^+^ T cells, this study aimed to further elucidate the sustained regulatory influence of MP‐12C on CD8^+^ T cell activation and its augmenting effect on effector functions. To achieve this, a co‐culture system utilizing tumor cell culture supernatant and PBMC was used for subsequent validation. CD137 is a member of the TNF receptor superfamily and is a crucial costimulatory molecule for CD8^+^ T cell activation. Its upregulation can amplify co‐stimulatory signals to T cells, markedly improving the proliferative capacity, survival, and cytotoxic function of CD8^+^ T cells, and is a fundamental metric for assessing the strength and duration of CD8^+^ T cell activation. T24 cells were randomly allocated into three groups: control, CDDP, and MP‐12C. After the pharmacological intervention, the culture supernatants from each group were harvested; these supernatants were co‐cultured with PBMC suspensions from healthy donors for 48 h, and the positive CD137 expression ratio in CD3^+^CD8^+^ T cells was assessed by flow cytometry (gating strategy: CD3^+^CD8^+^ T cells). The results indicated that, in comparison to the control group, the positive ratio of CD137 in CD3^+^CD8^+^ T cells in the CDDP group exhibited no significant change, and the difference lacked statistical significance; conversely, when compared to both the control and CDDP groups, the positive ratio of CD137 in CD3^+^CD8^+^ T cells in the MP‐12C group demonstrated a significant increase. The findings indicated that MP‐12C therapy initiated early activation of CD8^+^ T cells and sustained their activation by upregulating the co‐stimulatory molecule CD137, thereby augmenting their antitumor efficacy (Figure 8E,J).

It was investigated whether the mature effector killing functions of CD8^+^ T cells could be affected by MP‐12C, given previous findings that MP‐12C significantly induced both early and sustained activation of CD8^+^ T cells. The positive expression ratio of GZMB in CD3^+^CD8^+^ T cells was assessed using flow cytometry, with the gating strategy focused on CD3^+^CD8^+^ T cells. GZMB is a crucial effector molecule for cytotoxic T lymphocytes (CTL) that facilitates target cell apoptosis. Its expression level is a fundamental determinant of CD8^+^ T cell differentiation into mature effector CTLs and the attainment of direct antitumor cytotoxicity, thus serving as a benchmark for assessing T cell effector function. The findings indicated that, in comparison to the control group, the positive proportion of GZMB in CD3^+^CD8^+^ T cells of the CDDP group exhibited no significant change, and the difference lacked statistical significance; conversely, when compared to both the control and CDDP groups, the positive proportion of GZMB in CD3^+^CD8^+^ T cells of the MP‐12C group demonstrated a highly significant increase (Figure 8F,K).

According to the above results, MP‐12C not only activated CD8^+^ T cells but also significantly facilitated their differentiation into effector CTLs, augmented their primary cytotoxic function, and ultimately elicited an effective antitumor immune response against bladder cancer.

### 2.14 MP‐12C Remodels the Immune Microenvironment of Bladder Cancer In Vivo

Following the previously mentioned in vitro experiments that established MP‐12C's capability to induce ICD in bladder cancer cells, activate the cGAS‐STING pathway, and facilitate the activation and differentiation of CD8^+^ T cells into effector CTLs, the regulation of the antitumor immune response by MP‐12C in the in vivo physiological microenvironment was investigated. The experimental groups and administration schedule were as follows: control, CDDP (2.5 mg platinum/kg), and MP‐12C (2.5 mg platinum/kg) groups, each administered once every three days for a total of three administrations. After the administration of the designated pharmacological agents, the tumor tissues of the mice were excised to generate single‐cell suspensions, and changes in the proportions of immune cell subsets within the tumor tissues were assessed via flow cytometry (the gating strategy was established: CD80^+^CD86^+^dendritic cells (DCs) were identified within the CD11c^+^ cell population, and CD8^+^ T cells were identified within the CD3^+^ cell population).

DCs possess the most robust immune functionality and serve as the central nexus linking the immunogenic demise of tumor cells, the cGAS‐STING pathway, and targeted activation of T cells. The CD11c^+^CD80^+^CD86^+^ phenotype is a crucial marker of DC development. Mature DCs can effectively deliver tumor antigens and activate naïve T cells, thereby initiating targeted antitumor immune responses. The flow cytometry results indicated that, relative to the control group, the proportion of CD80^+^CD86^+^ mature DCs within the CD11c^+^ cell population in the tumor tissues of the CDDP group was markedly reduced. Conversely, compared to both the control and CDDP groups, the proportion of mature DCs in the tumor tissues of the MP‐12C group was significantly elevated, thereby confirming that MP‐12C effectively enhanced the maturation of tumor‐infiltrating DCs in vivo and improved their antigen‐presenting capacity (Figure 8G,L).

The infiltration of CD3^+^CD8^+^ T cells into tumor tissues is a critical determinant of the success of antitumor immune responses and an essential predictor of immunotherapy effectiveness. The results indicated that the infiltration proportion of CD8^+^ T cells within the CD3^+^ T cell population in tumor tissues from the CDDP group was significantly higher than in the control group. Furthermore, relative to both the control and CDDP groups, the proportion of CD3^+^CD8^+^ T cells in the tumor tissues of the MP‐12C group was markedly increased, thereby confirming that MP‐12C significantly enhanced the recruitment and infiltration of effector CD8^+^ T cells into tumor tissues and effectively activated antitumor adaptive immunity (Figure 8H,M).

Based on the above results, MP‐12C remodeled the immune microenvironment of bladder cancer in vivo by enhancing DC maturation and facilitating CD8^+^ T cell infiltration.

## 3 Discussion

Previous research has established that co‐administration of MT with CDDP can augment therapeutic efficacy, particularly in human lung adenocarcinoma [18], cervical cancer [26], and bladder cancer [27]. Our study also verified that a low dose of MT significantly augmented CDDP cytotoxicity in bladder cancer cells. However, when co‐administered, their pharmacokinetic profiles are mismatched, making it difficult to achieve synchronous delivery to tumor tissues. Consequently, a novel platinum(IV) prodrug, MP‐12C, which conjugates CDDP and MT into a single entity, was developed to facilitate the simultaneous release of both active constituents within tumor cells, thereby producing a synergistic sensitizing effect. MP‐12C markedly enhanced the platinum concentration in tumor cells. This is ascribed to its lipid‐soluble composition, which facilitates passive diffusion. This dual mechanism significantly enhanced the antitumor potency of MP‐12C. In multiple bladder cancer cell lines, the proliferation‐inhibitory efficacy of MP‐12C significantly surpassed that of CDDP and mitomycin C. CDDP resistance is the primary factor contributing to chemotherapy failure in bladder cancer, with mechanisms primarily involving reduced intracellular drug accumulation due to the overexpression of drug efflux pumps, augmented DNA damage repair capabilities, and activation of anti‐apoptotic pathways [28]. The T24/DDP cell line, with a resistance index of 6.6, had significant sensitivity to MP‐12C, with an IC_50_ of about 0.008 µM. This indicates that MP‐12C effectively overcomes cisplatin resistance and holds substantial promise for treating patients with recurrent and resistant malignancies.

The in vivo trials further validated the excellent efficacy and low toxicity profile of intravenously administered MP‐12C. In the T24 subcutaneous tumor model, the tumor suppression rate of MP‐12C was 67.8% at an equivalent platinum dosage, markedly surpassing the 39.4% observed with CDDP. The body weight of mice in the MP‐12C cohort showed no significant reduction, with no fatalities, whereas the CDDP group exhibited weight loss and mortality. In the Luc‐MB49 in situ bladder cancer model, the tumor suppression rate with intravenous MP‐12C reached 97.3%, far surpassing the 51.9% achieved with CDDP. Tissue distribution analysis indicated that platinum accumulation in the tumors of the MP‐12C group was much greater than in the CDDP group, whereas accumulation in the kidneys was dramatically reduced. The platinum(IV) configuration of MP‐12C is stable in circulation, minimizing exposure to normal tissues, whereas its lipophilicity facilitates tumor targeting and activation solely within cells. Hematological analysis, biochemical assays, and histological staining confirmed that CDDP induced bone marrow suppression, as well as hepatic and renal injury, whereas MP‐12C was safe.

In addition to intravenous administration, MP‐12C showed exceptional potential for intravesical instillation therapy, which is the standard of care for NMIBC. The total blood platinum exposure following MP‐12C bladder instillation for intravesical therapy of NMIBC was significantly lower than that with CDDP, resulting in reduced systemic toxicity. In the in situ tumor model, the tumor suppression rate of low‐dose MP‐12C was 91.1%, surpassing that of CDDP (61.7%) and mitomycin C (70.4%), and the tumor suppression rate of high‐dose MP‐12C reached 99.0%. In the high‐dose CDDP cohort, all mice died after two instillations; however, no fatalities occurred in the MP‐12C group. Body weight, hematological parameters, and hepatic and renal functions were all within normal limits. The benefits of MP‐12C include improved urothelial penetration due to its high lipid solubility and minimal systemic absorption. Its high lipophilicity (improved urothelial penetration) and minimal systemic absorption align with the unmet clinical requirements for intravesical NMIBC instillation therapy. In the BBN spontaneous bladder cancer model, the intravesical instillation of MP‐12C significantly reduced the tumor T stage and inhibited the progression from NMIBC to MIBC, thereby addressing the high progression rates of current intravesical instillation therapies.

Previous research has established that low‐dose MT can markedly augment the anti‐bladder cancer efficacy of CDDP; however, the precise molecular mechanism by which it facilitates CDDP chemosensitization remains poorly elucidated. Consequently, transcriptome sequencing was used to perform GO and KEGG enrichment analyses, revealing that MP‐12C treatment specifically and significantly modulated biological processes and signaling pathways associated with the extracellular matrix. The core was enriched in the ECM‐receptor interaction pathway, whereas CDDP therapy did not substantially enhance ECM‐related entries and pathways. This outcome indicates that the conventional antitumor mechanism of CDDP primarily depends on its capacity to induce DNA damage and does not significantly influence ECM remodeling in the tumor microenvironment. Conventional cisplatin fails to address secondary resistance driven by aberrant ECM deposition. In contrast, MP‐12C, through its association with the MT group, integrates the DNA‐damaging efficacy of CDDP with the ECM‐regulatory properties of MT, thereby facilitating chemosensitization via targeted ECM remodeling. GSEA further indicated that the ECM‐receptor interaction route was the most significantly altered signaling pathway following MP‐12C and CDDP treatment, which is the principal functional mechanism by which MT mediates CDDP chemosensitization. ELISA revealed that MT and MP‐12C reduced collagen VI secretion by T24 cells, with COL6A3, which encodes collagen VI, as the primary target. Reducing collagen VI release by T24 cells markedly enhanced their sensitivity to CDDP. *COL6A3* encodes the α3 chain of type VI collagen, which serves as the core domain of collagen VI and is intimately associated with malignant growth in various cancers. In bladder cancer, *COL6A3* is significantly expressed in bladder cancer tissues and is positively correlated with pathological grade, clinical stage, and poor patient prognosis [29]; however, its impact on the sensitivity of bladder cancer to CDDP chemotherapy and the underlying regulatory mechanisms remains unclear.

Transcription factors are key regulators of gene expression that bind to specific cis‐elements in gene promoters to control transcription. They are essential for the transcriptional control of malignancies during progression and for treatment resistance [30]. In this study, based on the screening approach of “specifically regulated by MT and MP‐12C, but unaffected by CDDP intervention,” and integrating transcriptome sequencing data and bioinformatics predictions, Transcription 4 (TCF4) was ultimately identified as the primary upstream transcription factor, through which MT and MP‐12C regulate the mRNA expression of *COL6A3*. TCF4 protein serves as a crucial downstream nuclear effector of the Wnt/β‐catenin signaling pathway, binding nuclear β‐catenin to form a transcriptional activation complex that initiates transcription of downstream target genes [31, 32]. Current research has established that the aberrant activation of the Wnt/β‐catenin/TCF4 signaling pathway is a fundamental mechanism driving the progression of bladder cancer malignancy and modulates the proliferation of bladder cancer cells [33]; similarly, in gastric [34] and neuroblastoma [35] cancers, elevated expression of TCF4 protein has been associated with resistance to chemotherapy. MT and MP‐12C suppress the transcriptional expression of the target gene COL6A3 by downregulating the transcription factor TCF4, thereby reducing collagen VI release by T24 cells and facilitating the chemosensitizing action of CDDP. This mechanism elucidates the primary advantage of MP‐12C over conventional CDDP: MP‐12C not only induces cytotoxicity via platinum moieties but also targets the *TCF4*/*COL6A3* axis through the MT moiety, reduces collagen VI secretion by T24 cells, enhances sensitivity to CDDP, and accomplishes the dual objectives of tumor eradication and chemosensitization.

Chemotherapy resistance [36], limited treatment response rates, and poor prognosis [37] remain significant issues in clinical practice. The immunosuppressive microenvironment of tumors is the primary factor driving the progression and treatment resistance of bladder cancer, and modifying the antitumor immune milieu is crucial for enhancing therapeutic efficacy. In this study, MP‐12C‐induced ICD was significantly more potent than CDDP‐induced ICD, effectively augmenting the immunogenicity of bladder cancer cells and laying the foundation for subsequent antitumor immune responses. This is the key advantage of MP‐12C over conventional platinum agents. MP‐12C consistently activates the cGAS‐STING pathway across diverse bladder cancer cell lines, whereas CDDP's efficacy is cell line‐specific, making MP‐12C applicable to a broader demographic than CDDP. The results of in vitro experiments indicated that MP‐12C significantly upregulated the early activation marker CD69, co‐stimulatory molecule CD137, and effector molecule GZMB in CD8^+^ T cells, thereby facilitating their differentiation into effector CTLs with mature cytotoxic functions. In contrast, although CDDP can directly induce tumor cell death, it fails to effectively promote the differentiation of CD8^+^ T cells into effector cells and may even inhibit antitumor immunity due to its nonspecific toxicity to immune cells. This is a fundamental immunological advantage of MP‐12C. The results of in vivo experiments demonstrated that MP‐12C significantly increased the proportion of mature DCs (CD11c^+^CD80^+^CD86^+^) in tumor tissues, whereas CDDP reduced it. This indicates that CDDP exhibits non‐specific toxicity toward DCs, impairing their maturation and antigen‐presentation capabilities and consequently hindering the initiation of the antitumor immune response. Simultaneously, MP‐12C markedly increased CD3^+^CD8^+^ T cell infiltration within the tumors, whereas the impact of CDDP was minimal. These findings unequivocally illustrate the fundamental advantages of MP‐12C over conventional platinum‐based therapies and establish a robust foundation for its clinical application.

This study has several limitations: (1) The pharmacokinetic data of MP‐12C needs further improvement. (2) There may be room for further optimization of clinical intravesical instillation protocols, such as intravesical instillation retention time and dosing frequency. (3) The kinetic measurement of Pt(IV) reduction and the direct correlation between reduction rate and biological activity under different GSH concentrations and pH values in complex cellular systems have not been explored. (4) The correlation between alkyl chain length and reduction rate was not explored, leading to an incomplete structure‐activity relationship analysis. (5) The mechanism by which MP‐12C induces ICD and activates the cGAS‐STING pathway, as well as the relationship between ICD and cGAS‐STING pathway activation, deserve further exploration.

In conclusion, a novel series of melatplatin(IV) prodrugs was developed, and MP‐12C was identified as a lead candidate that simultaneously has excellent anti‐cancer efficacy, overcomes cisplatin resistance, reduces systemic toxicity, and remodels the tumor immune microenvironment by inducing ICD and dual targeting of TCF4/COL6A3‐mediated ECM remodeling and the cGAS‐STING pathway. This study provides a promising therapeutic strategy and a preclinical foundation for the treatment of NMIBC and MIBC (Scheme 1).

## 4 Methods

### 4.1 Purity of Target Compounds

The purities of all the compounds were determined by HPLC. HPLC analysis was carried out on a Shimadzu Prominence HPLC system equipped with a Venusil XBP‐C18 column (5 µm, 150 Å, 250 mm × 4.6 mm). The mobile phase was a methanol–water gradient (95:5, v/v, containing 1% formic acid), and the flow rate was 1.0 mL/min. The wavelength of the UV detector was set to 260 nm. The purity of the compounds was confirmed to be > 95% using HPLC. The mobile phase composition used for HPLC is shown in Table S1.

### 4.2 The Synthesis of Cis,Cis,Trans‐Diamminedichlorodi‐Hydroxoplatinum(IV) (Oxoplatin)

Oxoplatin was synthesized as described previously [38]. The CDDP (1 g, 3.33 mmol) was added to H_2_O_2_ (30% w/v, 6 mL) at 70°C for 6 h, then the bright yellow solution was cooled at 4°C overnight, and the yellow solid obtained by centrifugation was washed twice with ice water, ethanol, and ether, respectively. Yield: 80%. HR‐MS (m/z) calcd for Cl_2_H_9_N_2_O_2_Pt: [M + H]+, 334.9611; found: 334.2917.

### 4.3 The Synthesis of Compounds 1‒4

Compounds **1‒4** were synthesized as reported previously [22, 23], and the steps for synthesis and data are provided in the supporting information.

### 4.4 General Procedure for the Synthesis of Compounds 5‒12

A dry DMF solution (2 mL) was added to 1 equivalent of **1** or **2**, and the mixture was stirred for 5 min. Then, 2.5 equivalents of isocyanate were added and allowed to react for 5 h to obtain a clear green solution. The DMF was removed under reduced pressure. A small amount of dichloromethane was added to dissolve the product, which was then separated and purified by column chromatography using dichloromethane/methanol (10:1).

*cis, cis‐diamminedichloro‐{4‐[3‐(2‐acetamidoethyl)‐5‐methoxy‐1H‐indol‐1‐yl]‐4‐oxobuta‐noato}‐hexylcarbamoa‐toplatinum(IV) (* ***5*** *)*: Compound **3** (50 mg, 0.08 mmol), hexyl isocyanate (25.44 mg, 0.2 mmol). Yield: 35 mg (56.5%). ^1^H NMR (400 MHz, DMSO‐*d*_6_): δ 8.21 (d, *J* = 9.0 Hz, 1H), 8.06 (t, *J* = 5.6 Hz, 1H), 7.69 (s, 1H), 7.14 (d, *J* = 2.5 Hz, 1H), 6.92 (dd, *J* = 9.0, 2.5 Hz, 1H), 6.63 (s, 6H), 6.57 (m, 1H), 3.81 (s, 3H), 3.38 (d, *J* = 7.2 Hz, 2H), 3.11 (t, *J* = 6.5 Hz, 2H), 2.88 (dd, *J* = 12.6, 6.3 Hz, 2H), 2.78 (t, *J* = 7.1 Hz, 2H), 2.72 (t, *J* = 6.2 Hz, 2H), 1.81 (s, 3H), 1.34 (d, *J* = 8.2 Hz, 2H), 1.26 (s, 6H), and 0.85 (t, *J* = 6.8 Hz, 3H). ^13^C NMR (101 MHz, DMSO‐*d*_6_): δ 179.48, 170.43, 169.20, 163.85, 155.80, 131.39, 129.82, 123.56, 119.07, 116.77, 112.86, 101.82, 55.32, 40.95, 38.17, 31.10, 30.74, 29.78, 26.09, 24.79, 22.67, 22.09, and 13.94. HR‐MS calculated for C_24_H_39_Cl_2_N_5_O_7_Pt (M + H)^+^, 776.5880; found: 776.1926.

*cis, cis‐diamminedichloro‐{4‐[3‐(2‐acetamidoethyl)‐5‐methoxy‐1H‐indol‐1‐yl]‐4‐oxobuta‐noato}‐octylcarbamoat‐oplatinum(IV) (* ***6***): Compound **3** (50 mg, 0.08 mmol), octyl isocyanate (31.05 mg, 0.2 mmol). Yield: 29.8 mg (46.4%). ^1^H NMR (400 MHz, DMSO‐*d*_6_): δ 8.21 (d, *J* = 8.8 Hz, 1H), 8.05 (s, 1H), 7.69 (s, 1H), 7.14 (s, 1H), 6.92 (d, *J* = 8.3 Hz, 1H), 6.63 (s, 6H), 6.56 (m, 1H), 3.81 (s, 3H), 3.36 (s, 2H), 3.12 (s, 2H), 2.88 (d, *J* = 5.3 Hz, 2H), 2.78 (t, *J* = 6.5 Hz, 2H), 2.73 (s, 2H), 1.82 (s, 3H), 1.33 (s, 2H), 1.23 (s, 10H), and 0.85 (d, *J* = 6.4 Hz, 3H). ^13^C NMR (101 MHz, DMSO‐*d*_6_): δ 179.48, 170.42, 169.20, 163.86, 155.80, 131.39, 129.82, 123.56, 119.07, 116.76, 112.86, 101.82, 55.32, 40.96, 38.17, 31.25, 30.75, 29.83, 28.78, 28.71, 26.44, 24.79, 22.67, 22.09, and 13.95. HR‐MS calculated for C_26_H_43_Cl_2_N_5_O_7_Pt (M + H)^+^, 804.6420; found: 804.2245.

*cis, cis‐diamminedichloro‐{4‐[3‐(2‐acetamidoethyl)‐5‐methoxy‐1H‐indol‐1‐yl]‐4‐oxobutanoato}‐dodecylcarbamo‐atoplatinum(IV) (* ***7***): Compound **3** (50 mg, 0.08 mmol), hexadecyl isocyanate (42.27 mg, 0.2 mmol). Yield: 33 mg (48.1%). ^1^H NMR (400 MHz, DMSO‐*d*_6_): δ 8.21 (d, *J* = 8.9 Hz, 1H), 8.05 (s, 1H), 7.69 (s, 1H), 7.14 (s, 1H), 6.92 (dd, 1H), 6.63 (s, 6H), 6.56 (m, 1H), 3.81 (s, 3H), 3.37 (s, 2H), 3.12 (s, 2H), 2.88 (d, *J* = 4.9 Hz, 2H), 2.79 (t, *J* = 6.5 Hz, 2H), 2.73 (s, 2H), 1.82 (s, 3H), 1.33 (s, 2H), 1.23 (s, 18H), and 0.85 (s, 3H). ^13^C NMR (101 MHz, DMSO‐*d*_6_): δ 179.48, 170.42, 169.20, 163.85, 155.80, 131.39, 129.82, 123.55, 119.06, 116.76, 112.85, 101.81, 55.31, 40.96, 38.17, 31.28, 30.74, 29.83, 29.07, 29.04, 29.02, 28.90, 28.71, 26.45, 24.79, 22.67, 22.09, and 13.95. HR‐MS calculated for C_30_H_51_Cl_2_N_5_O_7_Pt (M + H)^+^, 860.7500; found: 860.2859.

cis, cis‐diamminedichloro‐{4‐[3‐(2‐acetamidoethyl)‐5‐methoxy‐1H‐indol‐1‐yl]‐4‐oxobuta‐noato}hexadecylcarba‐moatoplatinum(IV) (**8**): Compound **3** (50 mg, 0.08 mmol), dodecyl isocyanate (53.49 mg, 0.2 mmol). Yield: 40 mg (54.7%). ^1^H NMR (400 MHz, DMSO‐d_6_): δ 8.21 (d, J = 9.0 Hz, 1H), 8.03 (t, J = 5.6 Hz, 1H), 7.68 (s, 1H), 7.14 (d, J = 2.4 Hz, 1H), 6.92 (dd, J = 9.0, 2.5 Hz, 1H), 6.61 (s, 6H), 6.55 (d, J = 5.4 Hz, 1H), 3.81 (s, 3H), 3.44 – 3.34 (m, 2H), 3.12 (t, J = 6.5 Hz, 2H), 2.93 – 2.83 (m, 2H), 2.78 (t, J = 7.1 Hz, 2H), 2.72 (t, J = 6.3 Hz, 2H), 1.81 (s, 3H), 1.33 (s, 2H), 1.23 (s, 26H), and 0.85 (t, J = 6.7 Hz, 3H). ^13^C NMR (101 MHz, DMSO‐d_6_): δ 179.48, 170.42, 169.19, 163.85, 155.79, 131.39, 129.82, 123.56, 119.06, 116.76, 112.85, 101.81, 55.30, 40.96, 38.16, 31.27, 30.72, 29.83, 29.04, 28.98, 28.90, 28.69, 26.45, 24.79, 22.67, 22.08, and 13.94. HR‐MS calculated for C_34_H_59_Cl_2_N_5_O_7_Pt (M + H)^+^, 916.8580; found: 916.3496.

*cis, cis‐diamminedichloro‐{5‐[3‐(2‐acetamidoethyl)‐5‐methoxy‐1H‐indol‐1‐yl]‐5‐oxopent‐anoato}‐hexylcarbamo‐atoplatinum(IV) (* ***9***): Compound **4** (53 mg, 0.08 mmol), hexyl isocyanate (25.44 mg, 0.2 mmol). Yield: 30 mg (47.6%). ^1^H NMR (400 MHz, DMSO‐*d*_6_): δ 8.22 (d, *J* = 8.9 Hz, 1H), 8.04 (t, *J* = 5.2 Hz, 1H), 7.69 (s, 1H), 7.14 (d, *J* = 1.9 Hz, 1H), 6.92 (dd, *J* = 8.9, 2.0 Hz, 1H), 6.67 (s, 6H), 6.53 (s, 1H), 3.81 (s, 3H), 3.39 (s, 2H), 3.02 (t, *J* = 7.1 Hz, 2H), 2.89 (d, *J* = 5.1 Hz, 2H), 2.78 (t, *J* = 7.0 Hz, 2H), 2.37 (t, *J* = 6.6 Hz, 2H), 1.92 – 1.83 (m, 2H), 1.82 (s, 3H), 1.35 (d, *J* = 5.9 Hz, 2H), 1.23 (s, 6H), and 0.86 (t, *J* = 6.6 Hz, 3H). ^13^C NMR (101 MHz, DMSO‐*d*_6_): δ 179.99, 171.21, 169.23, 163.99, 155.79, 131.39, 129.74, 123.84, 118.91, 116.71, 112.86, 101.83, 55.32, 40.95, 38.25, 34.53, 33.99, 31.10, 29.78, 26.09, 24.81, 22.69, 22.09, 20.74, and 13.95. HR‐MS calculated for C_25_H_41_Cl_2_N_5_O_7_Pt (M + H)^+^, 790.6150; found: 790.2081.

*cis, cis‐diamminedichloro‐{5‐[3‐(2‐acetamidoethyl)‐5‐methoxy‐1H‐indol‐1‐yl]‐5‐oxopent‐anoato}octylcarbamoat‐oplatinum(IV) (* ***10***): Compound **4** (53 mg, 0.08 mmol), octyl isocyanate (31.05 mg, 0.2 mmol). Yield: 21 mg (32.2%). ^1^H NMR (400 MHz, DMSO‐*d*_6_): δ 8.22 (d, *J* = 8.0 Hz, 1H), 8.04 (s, 1H), 7.69 (s, 1H), 7.14 (s, 1H), 6.92 (d, *J* = 7.6 Hz, 1H), 6.60 (d, *J* = 54.7 Hz, 7H), 3.81 (s, 3H), 3.36 (s, 2H), 3.02 (s, 2H), 2.89 (dd, *J* = 1.0, 0.4 Hz, 2H), 2.78 (s, 2H), 2.37 (s, 2H), 1.86 (s, 2H), 1.82 (s, 3H), 1.34 (s, 2H), 1.23 (s, 10H), and 0.86 (s, 3H). ^13^C NMR (101 MHz, DMSO‐*d*_6_): δ 180.00, 171.21, 169.23, 163.97, 155.79, 131.39, 129.74, 123.84, 118.91, 116.72, 112.86, 101.83, 55.32, 40.94, 38.26, 34.53, 34.00, 31.26, 29.83, 28.85 28.72, 26.45, 24.81, 22.69, 22.09, 20.74, and 13.96. HR‐MS calculated for C_26_H_43_Cl_2_N_5_O_7_Pt (M + H)^+^, 818.6690; found: 818.2401.

cis, cis‐diamminedichloro‐{5‐[3‐(2‐acetamidoethyl)‐5‐methoxy‐1H‐indol‐1‐yl]‐5‐oxopent‐anoato}‐dodecylcarba‐moatoplatinum(IV) (**11**): Compound **4** (53 mg, 0.08 mmol), dodecyl isocyanate (53.49 mg, 0.2 mmol). Yield: 36 mg (51.6%). ^1^H NMR (400 MHz, DMSO‐d_6_): δ 8.22 (d, J = 8.8 Hz, 1H), 8.05 (s, 1H), 7.69 (s, 1H), 7.14 (s, 1H), 6.92 (d, J = 8.3 Hz, 1H), 6.68 (s, 6H), 6.53 (s, 1H), 3.81 (s, 3H), 3.36 (s, 2H), 3.02 (t, J = 6.2 Hz, 2H), 2.88 (d, J = 3.8 Hz, 2H), 2.78 (t, J = 6.3 Hz, 2H), 2.37 (s, 2H), 1.92–1.84 (m, 2H), 1.82 (s, 3H), 1.34 (s, 2H), 1.23 (s, 18H), and 0.85 (d, J = 6.4 Hz, 3H). ^13^C NMR (101 MHz, DMSO‐d_6_): δ 180.01, 171.20, 169.22, 163.98, 155.79, 131.39, 129.75, 123.84, 118.91, 116.71, 112.85, 101.83, 55.31, 40.95, 38.26, 34.53, 34.00, 31.28, 29.83, 29.07, 29.04, 29.02, 28.90, 28.71, 26.45, 24.81, 22.68, 22.08, 20.75, and 13.94. HR‐MS calculated for C_31_H_53_Cl_2_N_5_O_7_Pt (M + H)^+^, 874.7770; found: 874.3028.

cis, cis‐diamminedichloro‐{5‐[3‐(2‐acetamidoethyl)‐5‐methoxy‐1H‐indol‐1‐yl]‐5‐oxopent‐anoato}‐hexadecylcarb‐amoatoplatinum(IV) (**12**): Compound **4** (53 mg, 0.08 mmol), hexadecyl isocyanate (53.49 mg, 0.2 mmol). Yield: 43 mg (57.9%). ^1^H NMR (400 MHz, DMSO‐d_6_): δ 8.22 (d, J = 8.9 Hz, 1H), 8.02 (t, J = 5.5 Hz, 1H), 7.68 (s, 1H), 7.14 (d, J = 2.0 Hz, 1H), 6.66 (s, 1H), 6.53 (m, 1H), 3.81 (s, 3H), 3.38 (d, J = 7.0 Hz, 2H), 3.02 (t, J = 7.2 Hz, 2H), 2.88 (d, J = 6.0 Hz, 2H), 2.78 (t, J = 7.2 Hz, 2H), 2.37 (t, J = 6.7 Hz, 2H), 1.90 – 1.84 (m, 2H), 1.82 (s, 3H), 1.33 (s, 2H), 1.23 (s, 26H), and 0.86 (d, J = 6.0 Hz, 3H). ^13^C NMR (101 MHz, DMSO‐d_6_): δ 180.00, 171.20, 169.21, 163.96, 155.79, 131.39, 129.75, 123.83, 118.91, 116.72, 112.85, 101.82, 55.31, 40.96, 38.26, 43.52, 34.00, 31.28, 29.84, 29.05, 29.00, 28.91, 28.70, 26.46, 24.82, 22.68, 22.09, 20.74, and 13.94. HR‐MS calculated for C_35_H_61_Cl_2_N_5_O_7_Pt (M + H)^+^, 930.8850; found: 930.3652.

### 4.5 Cell Culture

The human bladder cancer cell lines T24 (Cat. No. HTB‐4, RRID: CVCL_0554) and UM‐UC‐3 (Cat. No. CRL‐1749, RRID: CVCL_1783) were purchased from ATCC (USA). And the mouse bladder cancer cell line MB49 (Cat. No. M4‐0501, RRID: CVCL_7076) were purchased from OriCell Co., Ltd. (China). All cell lines were consistently grown in a temperature‐controlled incubator at 37°C under 5% CO_2_. The specific culture medium used for each cell line was as follows: T24 was cultured in RPMI‐1640 medium (Procell, PM150110) supplemented with 10% fetal bovine serum (FBS) (Procell, 164210); UM‐UC‐3 was cultured in MEM (Procell, PM150410) supplemented with 10% FBS; and MB49 was cultured in DMEM (Procell, PM150210) supplemented with 10% FBS. All culture media were supplemented with 100 U/mL penicillin‐streptomycin (Solarbio, P1400) to inhibit bacterial contamination.

### 4.6 Plate Colony Formation Assay

T24 cells in the logarithmic growth phase were inoculated at a density of 500‒1000 cells per well in a 6‐well plate and incubated overnight to facilitate cell adhesion. Subsequently, the appropriate medications were administered in accordance with the experimental treatment design, and the cells were cultivated for 7–10 days. Additionally, colony formation was observed. In the experiment to determine whether MT may enhance sensitivity to CDDP, the MT concentration was 100 µm, and the CDDP concentration was 0.5 µm. To evaluate the in vitro anti‐cancer efficacy of MP‐12C relative to that of CDDP and oxoplatin, the concentrations of oxoplatin, CDDP, and MP‐12C were uniformly set to 0.05 µm. To achieve optimal colony size, the growth medium was discarded, and the cells were rinsed twice with PBS, then fixed at room temperature for 30 min with 4% paraformaldehyde (Solarbio, P1110). Subsequently, 2 mL of 0.1% crystal violet (Solarbio, G1063) staining solution was added, and the cells were stained at ambient temperature in the dark for 30 min. The surplus staining solution was carefully rinsed with running water, and the samples were allowed to air‐dry naturally. Subsequently, photos of the complete wells were acquired, and the colony number and clone production rate were quantified using ImageJ software.

### 4.7 EdU Cell Proliferation Assay

T24 cells in logarithmic growth phase were seeded into 24‐well plates. Following overnight adhesion, when the degree of cell fusion reached 40%‒50%, the cells were treated with drugs corresponding to their respective groups for 24 h. In the experiment to determine whether MT may enhance the efficacy of CDDP, the MT concentration was 100 µm, whereas the CDDP concentration was 0.5 µm. For the in vitro comparison of the anti‐cancer activity of MP‐12C and CDDP, both agents were used at a concentration of 0.05 µm. Subsequently, 10 µmol/L EdU working solution (diluted with complete medium) was added to each well and incubated in the dark for 3 h, following the protocol of the EdU reagent kit (Beyotime, C0075). The culture medium was removed, and the cells were washed with PBS and fixed with 4% paraformaldehyde at room temperature for 15 min. Subsequently, cells were washed with PBS and permeabilized with 0.3% Triton X‐100 (Solarbio, T8200) in PBS for 15 min. The Click reaction solution was prepared according to the manufacturer's instructions. A suitable volume of the reaction solution was dispensed into each well and incubated at ambient temperature in the dark for 30 min. After washing with PBS, Hoechst 33342 (diluted 1:1000) was added for 10 min at ambient temperature in the dark. Images were collected using an inverted fluorescence microscope; the number of EdU‐positive cells and the total cell count were counted in ImageJ, and the proliferation rate was calculated.

### 4.8 CCK‐8 and MTT Cell Viability Assay

Logarithmically growing human (T24 and UM‐UC‐3) and murine bladder cancer cells (MB49) were seeded into 96‐well plates at a suitable density. Following overnight incubation, complete medium with gradient doses of drugs (100 µL per well) was administered. Three replicate wells were established for each concentration, with all drug groups having a maximum concentration of 100 µm, diluted continuously across a total of 18 concentration gradients. Following a 72‐h culture period, the samples were processed according to the instructions of the CCK8‐Kit (Bimake, B34304) or MTT‐Kit (Beyotime, C0009S). The absorbance (OD) at 450 or 570 nm was quantified using an enzyme reader. A dose‐effect curve was generated using GraphPad Prism software (version 10.0), and the half‐maximal inhibitory concentration (IC_50_) for each medication across various cell lines was determined.

### 4.9 Intracellular Platinum Uptake

T24 cells in the logarithmic growth phase were seeded into 6‐well plates. Following overnight adherence, a complete medium supplemented with 10 µm CDDP, oxoplatin, or MP‐12C was administered for 4 h. The drug‐containing media were discarded and rinsed twice with PBS at ambient temperature. The cells were subjected to trypsin digestion, resuspended in PBS, counted, adjusted to 1 × 10^6^ cells per tube, and centrifuged at 3000 rpm at 4°C. The cell pellet was then collected and freeze‐dried. Concentrated nitric acid (200 µL) was added, and the sample was heated at 70°C for 4 h until completely digested. The platinum content was measured with a PerkinElmer NexION 1000 ICP‐MS.

### 4.10 Apoptosis Assay

T24 cells in the logarithmic growth phase were seeded into 6‐well plates and allowed to adhere overnight. Subsequently, a medium containing 1 µm of the medication was added, and the cells were treated for 24 h. The drug‐containing medium was removed, and the cells were rinsed twice with pre‐cooled PBS. The cells were then subjected to trypsin digestion and collected. The procedure was performed according to the guidelines of the Annexin V‐FITC/PI Cell Apoptosis Kit (Elabscience, E‐CK‐A211), followed by flow cytometric analysis.

### 4.11 Immunofluorescence Staining

T24 cells in the logarithmic growth phase were seeded onto a confocal microplate. Following 4 h of treatment with 10 µm of the medication, the cells were fixed with 4% paraformaldehyde for 15 min, permeabilized with 0.25% Triton X‐100 for 15 min, and subsequently blocked with 5% FBS for 1 h. Following this, primary antibodies, diluted to 1:200 (γ‐H2AX, Servicebio, GB111841; CRT, Proteintech, 27298‐1‐AP; HMGB1, Proteintech, 10829‐1‐AP), were added and incubated overnight at 4°C. The cells were subsequently treated with fluorescently tagged secondary antibodies (1:200 dilution) at room temperature in the dark for 2 h. The cell nuclei were labeled with DAPI. Images were acquired using confocal microscopy.

### 4.12 Induction of T24/DDP Bladder Cancer Cells

T24/DDP bladder cancer cells were developed by gradually escalating the CDDP concentration and intermittent induction. Log‐phase, healthy human bladder cancer T24 parental cells were cultured in RPMI‐1640 media supplemented with 10% FBS and 1% penicillin–streptomycin double antibiotics at 37°C, 5% CO_2_, and saturated humidity within a constant temperature incubator. Induction was initiated when cell confluence reached 60%–70%. We used 1/20 of the IC_50_ value of CDDP for parental T24 cells as the initial induction concentration. The fresh drug‐infused medium was replaced every 2–3 days, and suspended necrotic cells were eliminated. Upon achieving stable proliferation of the surviving cells and reaching a confluence of 80%–90%, the cells were sequentially treated for three to five iterations at a fixed drug concentration, and the CDDP induction concentration was incrementally increased. The screening and passage procedures were repeated. After six months of sustained induction, a cell line capable of stable proliferation and transmission in a high‐concentration CDDP growth medium was acquired. The CCK‐8 assay was used to assess the cell proliferative activity, and the RI (resistance cell IC_50_/parental cell IC_50_) was determined. A stable resistant cell line, T24/DDP, was established when the RI exceeded 5, and the resistance exhibited no significant variations after three consecutive passages. In subsequent trials, resistant cells were maintained in full medium with the appropriate concentration of CDDP. Drug administration for two passes was stopped before the experiment to eliminate the influence of drug residues on the results.

### 4.13 Animal Care and Use

Female BALB/c nude mice (5 weeks old, 14–18 g body weight) and female C57BL/6 mice (5‐7 weeks old, 12–18 g body weight) were purchased from Beijing SiPeiFu Biotechnology Co., Ltd. (Beijing, China). All animals were housed under specific pathogen‐free conditions and in individually ventilated cages under controlled environmental conditions: temperature 22 ± 2°C, relative humidity 50 ± 10%, and a 12‐h light/dark cycle. Mice had free access to sterilized standard rodent chow and autoclaved drinking water. All mice were allowed a 7‐day acclimatization period before the start of experiments.

For group allocation, mice with comparable tumor volumes (subcutaneous CDX model) or bioluminescent ROI values (orthotopic models) or degree of hematuria (BBN models) were randomly assigned to treatment groups using a completely randomized design. The sample sizes for each experiment were as follows: CDX model (n = 5 mice per group), orthotopic model with intravenous administration (n = 4 mice per group), orthotopic model with intravesical instillation (n = 5 mice per group), BBN‐induced spontaneous model (n = 10 mice per group).

Humane endpoints were pre‐defined and strictly monitored throughout the study. Mice were euthanized immediately before the Institutional Animal Care and Use Committee (IACUC)‐approved 2000 mm^3^, experiencing weight loss exceeding 20%, or meeting other humane endpoint criteria.

### 4.14 CDX Model of Bladder Cancer

T24 cells in the logarithmic growth phase were resuspended in PBS and calibrated to a density of 1 × 10^8^ cells/mL. A 100‐µL cell suspension, containing 1 × 10^7^ cells, was injected subcutaneously into the right flank of BALB/c nude mice. Upon reaching a tumor volume of 150–200 mm^3^, the mice were randomly assigned to treatment groups. Body weight was recorded every three days, and the long and short diameters of the tumor were measured to compute the volume (V = 1/2 × long diameter × short diameter^2^). At the end of the experiment, the mice were euthanized, the tumors were excised and weighed, and the tumor inhibition rate was determined. Tumor tissues and primary organs were preserved in 4% paraformaldehyde for histological analyses.

### 4.15 In Situ Model of Bladder Cancer

To create the fLuc‐MB49 cell line, MB49 cells were infected with a luciferase‐expressing lentivirus (Genechem, China) and subsequently selected with puromycin (2 µg/mL). Female C57BL/6 mice, aged six to eight weeks, were sedated, and their abdomens were incised to reveal the bladder. The cell suspension was injected into the bladder wall, and the abdominal wall was sutured. After the model was established, fluorescein potassium salt (Meilun, MB1834) (0.15 mg/g body weight) was administered intraperitoneally, and the ROI value was measured. The mice were randomly assigned to groups and subjected to treatment. The intravenous doses of CDDP and MP‐12C were 2.5 mg platinum/kg each. The doses for bladder instillation of CDDP and MP‐12C were 10 and 20 µmol/kg, respectively, while the dose of mitomycin C was 20 µmol/kg. This study involved observing the growth of in situ bladder cancer using small animal imaging. Upon completion of the experiment, blood was collected for routine blood and biochemical analyses, and tumors and major organs were dissected. Certain samples were designated for ICP‐MS analysis of platinum content, while the remainder were preserved for histological examination.

### 4.16 Detection of Total Blood Platinum Exposure

C57BL/6 mice aged six to eight weeks were selected. Post‐anesthesia, the relevant medications (CDDP and MP‐12C) were delivered via the urethra at a dose of 20 µmol/kg. After 1 h, blood was extracted. Whole blood (100 µL) was collected, combined with concentrated nitric acid, and digested at 70°C for 4 h. The solution was diluted, and the platinum content was analyzed using ICP‐MS.

### 4.17 Spontaneous BBN‐Induced Bladder Cancer Model

BBN (TCI, B0938) was diluted to 0.05% in an opaque drinking container and offered to C57BL/6 mice for unrestricted ingestion over 4 months. The BBN‐containing water was replaced every three days. Subsequently, the water was replaced with regular drinking water, and the mice were maintained on this diet for an additional two weeks. Mice with tumors were evaluated using MRI and urine test strips, which indicated positive hematuria. The positive mice underwent intravesical perfusion treatment weekly for four consecutive weeks, followed by an additional two weeks of feeding after cessation of medication administration. Tumor samples were preserved in 4% paraformaldehyde for histological analysis. The slices were stained with HE, and the pathological stage was assessed.

### 4.18 Transcriptome Sequencing

T24 cells in the logarithmic growth phase were seeded into T25 cell culture flasks. Following overnight adherence, 2 µm CDDP or MP‐12C was added to the medium, and the cells were treated for 24 h. The drug‐containing medium was removed, and the cells were rinsed twice with pre‐cooled PBS. Total RNA was isolated from T24 cells using TRIzol reagent (Takara, 9108), followed by high‐throughput sequencing on the Illumina Novaseq 6000 platform (performed by Hangzhou Lianchuan Biotechnology, China). Genes exhibiting a fold change of at least 2 and a *q*‐value < 0.05 were classified as DEGs. Point diagrams illustrating several group differences were generated using Xiantaozi (https://www.xiantaozi.com/) to visualize the DEGs across the groups. Bioinformatics analysis was conducted using OmicStudio tools available at https://www.omicstudio.cn/tool. Transcriptome sequencing was performed with three biological replicates per group.

### 4.19 qRT‐PCR

Total RNA was isolated from cells using TRIzol reagent, and cDNA was generated using Prime Script RT Master Mix (Takara, RR036). qRT‐PCR was conducted using SYBR Green (Takara, RR820) to determine the mRNA expression levels. GAPDH served as the internal reference, and the relative expression level of the target gene was determined using the 2^−^ΔΔCt technique. The following primer sequences were used: *COL6A3* (forward: ATGAGGAAACATCGGCACTTG; reverse: GGGCATGAGTTGTAGGAAAGC) and *GAPDH* (forward: GGAGCGAGATCCCTCCAAAAT; reverse: GGCTGTTGTCATACTTCTCATGG).

### 4.20 Western Blotting Assay

T24 cells in the logarithmic growth phase were seeded into T25 cell culture flasks and allowed to adhere overnight. Consequently, suitable treatments were administered. In the experiment investigating the impact of various drugs on TCF4 protein expression, distinct T24 samples were individually treated with complete media containing 2 µm CDDP, 2 µm MP‐12C, and 100 µm MT for 24 h. In the experiment investigating whether TCF4 overexpression can reinstate MT protein expression in TCF4 and COL6A3, T24 cells were initially infected with a TCF4 overexpression lentivirus (Genechem) and subsequently subjected to comprehensive screening with puromycin (2 µg/mL) (Solarbio, P8230). Following this, a complete medium containing 100 µm MT was added to both normal T24 cells and T24 cells overexpressing TCF4 for 24 h. The medium was removed, the cells were rinsed twice with pre‐chilled PBS, and RIPA lysis buffer (Beyotime, P0013B) with PMSF (Beyotime, ST506) and phosphatase inhibitors (Beyotime, P1081) was added for ice‐cold lysis for 30 min. Ultrasonic disruption was applied for 5 s, followed by a 10‐s pause, and repeated for 3 cycles. The supernatant was thereafter collected at 4°C and centrifuged at 12 000 rpm for 30 min. The protein concentration was quantified using the BCA technique (Beyotime, P0012) and standardized across various groups. The mixture was combined with 5×SDS loading buffer and denatured at 100°C for 10 min. Proteins were isolated by SDS‐PAGE and subsequently transferred to a PVDF membrane (Millipore, 3010040001). The PVDF membrane was treated overnight at 4°C with the respective primary antibodies (cGAS, ABclonal, A8335; Sting, CST, 13647; P‐sting, Affinity, AF7416; IRF3, Proteintech, 11312‐1‐AP; P‐IRF3, Immunoway, YP0326; COL6A3, Proteintech, 83010‐1‐RR; TCF4, Proteintech, 22337‐1‐AP; GAPDH, Proteintech, 60004‐1‐Ig). The secondary antibody conjugated with HRP was incubated at ambient temperature for 1 h, followed by the application of an ECL chemiluminescent solution (Beyotime, P0018S) for detection.

### 4.21 Bioinformatics Analysis

Transcription factor‐binding sites were predicted using the JASPAR database. Promoter sequences were obtained from NCBI (https://www.ncbi.nlm.nih.gov/), and primers were created using the Primer3Plus tool (https://www.primer3plus.com/). Putative transcription factors were identified by cross‐referencing the ChEA3 database within the TF_Target_Finder database (https://jingle.shinyapps.io/TF_Target_Finder/) using the differential expression patterns of the transcriptome. The relationship between the mRNA expression levels of *TCF4* and *COL6A3* was examined at https://www.xiantaozi.com/, utilizing transcriptome data from the TCGA‐BLCA cohort.

### 4.22 ChIP

T24 cells in the logarithmic growth phase were inoculated into 10 cm culture dishes. In accordance with the ChIP kit instructions (Thermo Fisher, 26157), the cells were cross‐linked using 1% formaldehyde at ambient temperature for 10 min, after which glycine was added to halt the process. PBS containing the cocktail inhibitor was added to the dish, and the cells were harvested. Cell nuclei were isolated and subjected to ultrasonic disruption to liberate soluble chromatin fragments. After centrifugation, the supernatant was collected, and 20 µg of target antibodies (TCF4), anti‐RNA polymerase II (positive control), or normal rabbit IgG (negative control) were subsequently added. The mixture was incubated overnight at 4°C. Protein A/G magnetic beads were incubated for 2 h, then rinsed with washing buffers I and II. The elution buffer was introduced, and the cross‐linking was undone at 65°C. Following proteinase K digestion, the DNA was purified using a DNA purification column. The enrichment of the target region was assessed by qRT‐PCR, using the input as an internal reference to calculate the fold enrichment.

### 4.23 Dual Luciferase Reporter Gene Assay

The luciferase reporter plasmid was acquired from GeneChem (China). Cells in the logarithmic growth phase were seeded into 6‐well plates. Upon achieving a fusion rate of 50%‒60%, the firefly luciferase reporter plasmid was co‐transfected with the Renilla luciferase reference plasmid. Experiments were conducted in accordance with the Dual‐Luciferase Reporter Assay System (Beyotime, RG027) 48 h post‐transfection, and luciferase activity was measured using a multifunctional microplate reader.

### 4.24 Co‐Culture of Immune and Tumor Cells

Freshly anticoagulated healthy human blood was obtained (the procedure was ethically approved by the Ethics Committee of the Second Hospital of Tianjin Medical University) and incorporated with a human PBMC separation solution (Solarbio, P9011). We used density gradient centrifugation to isolate PBMC from human peripheral blood. The magnetic beads were activated and expanded at a CD3/CD28 (Invitrogen, 11161D) ratio of 1:1 for CD8^+^ T cells in X‐VIVO 15 complete medium (Lonza, USA) (including 500 IU/mL recombinant human IL‐2 (PeproTech, 200–02)). Cultivation was maintained for 7 days, and half of the medium was replaced every 2–3 days. On the seventh day, the magnetic beads were detached, and the cells were harvested. The tumor cell culture supernatant was gathered and subjected to drug treatment (both CDDP and MP‐12C at 2 µm for 24 h), followed by co‐culturing with CD8^+^ T cells for either 24 or 48 h. Then, Cell Activation Cocktail with Brefeldin A (Biolegend, 423303) was added, and the mixture was incubated at 37°C under 5% CO_2_ in a constant temperature incubator for 5.5 h. Subsequently, cells were collected for flow cytometric analysis.

### 4.25 Preparation of a Single‐Cell Suspension of Tumor Tissue‐Infiltrating Immune Cells

Under aseptic conditions, tumor tissue from mice was dissected, minced, and then introduced into Hank's solution containing 1.0 mg/mL collagenase IV (Solarbio, C8160), 0.1 mg/mL DNase I (Solarbio, D8071), and 0.1 mg/mL hyaluronidase (Solarbio, H8030). Following digestion at 37°C for 30 min, the slurry was filtered through a 70‐µm cell sieve and centrifuged to harvest the cells for flow cytometry labeling.

### 4.26 Flow Cytometry for Immune Cell Identification

Following the obstruction of Fc receptors using the Staining Buffer (Beijing 4A, FXP005) with Human TruStain FcX (Biolegend, 422301), the viability dye (1:100) and surface antibodies (anti‐human CD3 (Biolegend, 317306), CD8 (Biolegend, 300908); anti‐mouse CD3 (Biolegend, 100217), CD8 (Biolegend, 100706), CD11C (Biolegend, 117309), CD80 (Biolegend, 104734), CD86 (Biolegend, 105007)) were introduced, and the mixture was incubated in the dark on ice for 30 min. For intracellular staining, the disrupted membranes were stabilized using the eBioscience Foxp3/Transcription Factor Staining Buffer Set (Invitrogen, 00‐5523‐00), followed by the addition of anti‐human Granzyme B (Biolegend, 372212), CD69 (Biolegend, 310910), and CD137 (Biolegend, 309830) antibodies, and incubation at 4°C in the dark for 30 min. Samples were acquired on a flow cytometer, and the expression rates of activation markers were analyzed using the FlowJo software.

### 4.27 Ethics Statement

All animal experiments were approved by the IACUC of Tianjin Jinke Bona Biotechnology Co., LTD (approval no. GENINK‐20250121) and were conducted in accordance with the National Institutes of Health Guide for the Care and Use of Laboratory Animals. Collecting fresh anticoagulated healthy human blood and extracting PBMCs was ethically approved by the Ethics Committee of the Second Hospital of Tianjin Medical University (approval no. KY2025K337). Informed consent has been obtained from all healthy blood donors. This study did not involve any clinical trial in human patients, and therefore no Clinical Study Registration Number is required.

### 4.28 Preparation of Schematic Illustrations/Table of Contents (ToC) Figures

The Scheme 1/ToC figure was created as follows: first, 3D models were built using Cinema 4D / Blender; subsequently, rendering was performed with the Redshift / Cycles renderer; finally, image adjustment and compositing were carried out in Photoshop. The schematic illustrations figures in Figure 3A, Figure 4A, Figure 5A, and Figure 6A were created with BioGDP.com [39].

### 4.29 Statistical Analysis

Sample sizes (n) for each type of experiment are specified as follows: in cellular experiments (n = 3 biological replicates); in animal experiments (n = 3∼10 biological replicates). Data are expressed as mean ± standard deviation (SD). Prior to statistical analysis, normality of data was tested by Shapiro–Wilk test. For data that passed the normality test, statistical differences between two groups were evaluated by two‐sided Student's t‐test, whereas statistical differences among three or more groups were evaluated by two‐side one‐way analysis of variance (ANOVA) followed by Tukey post‐hoc test. For data that did not pass the normality test, statistical differences between two groups were evaluated by the two‐sided Mann‐Whitney U test, whereas statistical differences among three or more groups were evaluated by the Kruskal‐Wallis H test with Dunn's post‐hoc correction. A two‐sided significance level (α) of 0.05 was adopted for all statistical tests. *P* < 0.05 was considered statistically significant. All statistical analyses were conducted using GraphPad Prism 10.0. ^*^ *p* < 0.05, ^**^ *p* < 0.01, and ^***^ *p* < 0.001, denote statistically significant differences; “ns” indicates no significance.

## Author Contributions

L. Wang, J. Xu, and H. Hu contributed to conceptualization; L. Wang, X. Liu, T. Zhu, R. Liu, Z. Li, K. Du, K. Jia, C. Fu, Y. Lin, Z. Xue, Z. Zhang, C. Shen, Y. Qie, Z. Wu, Z. Liu, K. Liu, J. Xu, and H. Hu developed the methodology; L. Wang, R. Liu, J. Xu, and H. Hu performed the investigation; L. Wang, X. Liu, T. Zhu, Z. Su, R. Liu, K. Liu, J. Xu, and H. Hu carried out visualization; X. Liu, Z. Li, Z. Zhang, C. Shen, Y. Qie, Z. Wu, Z. Liu, K. Liu, J. Xu, and H. Hu supervised the work; C. Shen, X. Liu, K. Liu, J. Xu, and H. Hu acquired the funding; L. Wang, T. Zhu, and K. Liu wrote the original draft; and X. Liu, K. Liu, J. Xu, and H. Hu conducted reviewing and editing.

## Conflicts of Interest

The authors declare no conflicts of interest.

## Supporting information




**Supporting File**: advs77833‐sup‐0001‐SuppMat.docx.

## Data Availability

Data supporting the findings of this study are available from the corresponding author upon request.
