# Supplementary material for: Isocyanate‐Modified Melatplatin(IV) Enhances Antitumor Efficacy via TCF4/COL6A3‐Mediated Extracellular Matrix Remodeling and cGAS‐STING Pathway Activation in Bladder Cancer
Source: Adv Sci (Weinh). 2026 Sep 27:e77833. Online ahead of print. doi: 10.1002/advs.77833 (PMC13616365; doi:10.1002/advs.77833)
Supplement: Supplementary file 1 [file ADVS-9999-e77833-s001.docx]

**Figure S1.** HR-MS spectrum of *c*, *c*, *t*-[Pt(NH_3_)_2_Cl_2_(OH)_2_].

**Figure S2**. ^1^H-NMR spectrum of **1** in DMSO-*d*_6_.

**Figure S3**. ^13^C-NMR spectrum of **1** in DMSO-*d*_6_.

**Figure S4**. HR-MS of **1** in methanol.

| No. | Retention time | Peak area | Concentration (%) |
| --- | --- | --- | --- |
| 1 | 11.933 | 2022 | 0.066 |
| 2 | 12.212 | 2225621 | 98.574 |
| 3 | 14.126 | 16254 | 0.811 |
| 4 | 14.544 | 11002 | 0.549 |

**Figure S5**. HPLC characterization of **1**.

**Figure S6**. ^1^H-NMR spectrum of **2** in DMSO-*d*_6_.

**Figure S7**. ^13^C-NMR spectrum of **2** in DMSO-*d*_6_.

**Figure S8***.* HR-MS of **2** in methanol.

| No. | Retention time | Peak area | Concentration (%) |
| --- | --- | --- | --- |
| 1 | 12.883 | 9036 | 0.525 |
| 2 | 13.075 | 1308 | 0.076 |
| 3 | 13.425 | 1773 | 0.103 |
| 4 | 13.732 | 1695053 | 98.475 |
| 5 | 14.033 | 14132 | 0.821 |

**Figure S9***.* HPLC characterization of **2**.

**Figure S10**. ^1^H-NMR spectrum of **3** in DMSO-*d*_6_.

**Figure S11**. ^13^C-NMR spectrum of **3** in DMSO-*d*_6_.

**Figure S12**. HR-MS of **3** in methanol.

| No. | Retention time | Peak area | Concentration (%) |
| --- | --- | --- | --- |
| 1 | 12.017 | 6333 | 0.248 |
| 2 | 12.240 | 1539675 | 97.638 |
| 3 | 13.609 | 30012 | 2.114 |

**Figure S13***.* HPLC characterization of **3**.

**Figure S14**. ^1^H-NMR spectrum of **4** in DMSO-*d*_6_.

**Figure S15**. ^13^C-NMR spectrum of **4** in DMSO-*d*_6_.

**Figure S16**. HR-MS of **4** in methanol.

| No. | Retention time | Peak area | Concentration (%) |
| --- | --- | --- | --- |
| 1 | 12.557 | 5929 | 0.356 |
| 2 | 12.814 | 1259068 | 98.186 |
| 3 | 13.527 | 9963 | 0.788 |
| 4 | 16.605 | 6586 | 0.669 |

**Figure S17***.* HPLC characterization of **4**.

**Figure S18.** ^1^H-NMR spectrum of **5**.

**Figure S19.** ^13^C-NMR spectrum of **5**.

**Figure S20***.* HR-MS of **5**.

| No. | Retention time | Peak area | Concentration (%) |
| --- | --- | --- | --- |
| 1 | 12.692 | 2784 | 0.141 |
| 2 | 14.627 | 2184069 | 99.495 |
| 3 | 14.924 | 10894 | 0.365 |

**Figure S21***.* HPLC characterization of **5**.

**Figure S22.** ^1^H-NMR spectrum of **6**.

**Figure S23.** ^13^C-NMR spectrum of **6**.

**Figure S24***.* HR-MS of **6**.

| No. | Retention time | Peak area | Concentration (%) |
| --- | --- | --- | --- |
| 1 | 12.692 | 2886 | 0.226 |
| 2 | 14.039 | 8380 | 0.841 |
| 3 | 15.075 | 3424 | 0.200 |
| 4 | 15.291 | 1365226 | 98.760 |

**Figure S25***.* HPLC characterization of **6**.

**Figure S26.** ^1^H-NMR spectrum of **7**.

**Figure S27.** ^13^C-NMR spectrum of **7**.

**Figure S28***.* HR-MS of **7**.

| No. | Retention time | Peak area | Concentration (%) |
| --- | --- | --- | --- |
| 1 | 16.575 | 9430 | 0.185 |
| 2 | 16.926 | 3744242 | 99.815 |

**Figure S29***.* HPLC characterization of **7**.

**Figure S30.** ^1^H-NMR spectrum of **8**.

**Figure S31.** ^13^C-NMR spectrum of **8**.

**Figure S32***.* HR-MS of **8**.

| No. | Retention time | Peak area | Concentration (%) |
| --- | --- | --- | --- |
| 1 | 18.071 | 13241 | 0.421 |
| 2 | 20.085 | 1629370 | 99.272 |
| 3 | 21.369 | 7301 | 0.307 |

**Figure S33***.* HPLC characterization of **8**.

**Figure S34***.* ^1^H-NMR spectrum of **9**.

**Figure S35***.* ^13^C-NMR spectrum of **9**.

**Figure S36***.* HR-MS of **9**.

| No. | Retention time | Peak area | Concentration (%) |
| --- | --- | --- | --- |
| 1 | 14.342 | 7307 | 0.777 |
| 2 | 14.592 | 2 380 | 0.180 |
| 3 | 14.806 | 1297838 | 99.043 |

**Figure S37***.* HPLC characterization of **9**.

**Figure S38.** ^1^H-NMR spectrum of **10**.

**Figure S39.** ^13^C-NMR spectrum of **10**.

**Figure S40***.* HR-MS of **10**.

| No. | Retention time | Peak area | Concentration (%) |
| --- | --- | --- | --- |
| 1 | 13.511 | 34039 | 1.262 |
| 2 | 15.491 | 1600981 | 98.738 |

**Figure S41***.* HPLC characterization of **10**.

**Figure S42.** ^1^H-NMR spectrum of **11**.

**Figure S43.** ^13^C-NMR spectrum of **11**.

**Figure S44***.* HR-MS of **11**.

| No. | Retention time | Peak area | Concentration (%) |
| --- | --- | --- | --- |
| 1 | 13.567 | 2665 | 0.220 |
| 2 | 13.736 | 5153 | 0.581 |
| 3 | 15.924 | 2723 | 0.197 |
| 4 | 16.534 | 1177673 | 97.642 |
| 5 | 16.870 | 13606 | 1.359 |

**Figure S45***.* HPLC characterization of **11**.

**Figure S46.** ^1^H-NMR spectrum of **12**.

**Figure S47.** ^13^C-NMR spectrum of **12**.

**Figure S48***.* HR-MS of **12**.

| No. | Retention time | Peak area | Concentration (%) |
| --- | --- | --- | --- |
| 1 | 17.480 | 7938 | 0.498 |
| 2 | 19.309 | 1094389 | 99.502 |

**Figure S49***.* HPLC characterization of **12**.

**Figure S50.** Melatonin enhances CDDP sensitivity in T24 bladder cancer cells.

(A) The inhibitory effects of Melatonin, CDDP, and their mixture on the proliferation of T24 bladder cancer cells were assessed using a plate cloning experiment.

(B) Statistical evaluation of the area generated by the plate cloning experiment (n = 3).

(C) EdU Cell Proliferation Assay to assess the inhibitory impact of Melatonin, CDDP, and their combination on the proliferation of T24 bladder cancer cells.

(D) Statistical examination of the proportion of EdU proliferating cells to the total cell count (n = 3). Data are presented as mean ± SD. Statistical analysis was performed using one-way ANOVA followed by Tukey's post-hoc test (B,D).

**Figure S51.** Hematoxylin and eosin-stained pathological sections of the primary organs (heart, liver, spleen, lung, kidney) of mice following administration of several intravenous drugs.

**Figure S52.** The outcomes of hematological analysis and biochemical assessment of blood in each mouse following various treatments with bladder instillation drugs (n = 3). Data are presented as mean ± SD. Statistical analysis was performed using one-way ANOVA followed by Tukey's post-hoc test (except for HGB, AST). Statistical analysis was performed using Kruskal-Wallis H test with Dunn's post-hoc correction (HGB, AST). **P* < 0.05, ***P* < 0.01, ****P* < 0.001; ns, not significant.

**F****igure S53.** Hematoxylin and eosin-stained pathological sections of the primary organs (heart, liver, spleen, lung, kidney) of mice following administration of several bladder instillation drugs.

**Figure S54.**

(A) Gene Ontology Enrichment analysis of differentially expressed genes between the MP-12C group and the CDDP group.

(B) Enrichment analysis of differentially expressed genes between the Ctrl group and the CDDP group using the Kyoto Encyclopedia of Genes and Genomes.

(C) Gene Ontology Enrichment analysis of differentially expressed genes between the Ctrl group and the CDDP group.

**Figure S55.** Gene Set Enrichment Analysis of differentially expressed genes between the MP-12C group and the CDDP group.

**Figure S56.** Evaluation and expression assessment of pivotal genes in the ECM-receptor interaction pathway.

(A) A Venn diagram illustrating the intersection screening of differential genes across several groups discovered 13 pivotal genes associated with ECM-receptor interaction pathways.

(B) Expression heatmap of six collagen genes across several treatment groups.

**Figure S57.** Reduction of MP-12C in tumor cells at 100 μM after 4 h treatment by HPLC.

**Figure S58.** Rescue effect of collagen VI on CDDP and MP-12C cytotoxicity (n = 3). Data are presented as mean ± SD. Statistical analysis was performed using one-way ANOVA followed by Tukey's post-hoc test. **P* < 0.05, ***P* < 0.01, ****P* < 0.001; ns, not significant.

**Table S1.** Mobile Phase of HPLC Analyses for the Purities of All Target Compounds.

| Time (min) | A (water) | B (methanol) |
| --- | --- | --- |
| 0.01 | 95 | 5 |
| 10.00 | 5 | 95 |
| 25.00 | 5 | 95 |
